# Distinct neural representations of perceptual and numerical absence in the human brain

**DOI:** 10.7554/eLife.112088

**Published:** 2026-11-05

**Authors:** Benjy Barnett, Stephen M Fleming

**Affiliations:** 1 Department of Imaging Neuroscience, University College London London United Kingdom; 2 School of Psychological Sciences, Birkbeck, University of London London United Kingdom; 3 Department of Experimental Psychology and Institute of Cognitive Neuroscience, University College London London United Kingdom; 4 Max Planck UCL Centre for Computational Psychiatry and Ageing Research, University College London London United Kingdom; 5 Canadian Institute for Advanced Research (CIFAR), Brain, Mind and Consciousness Program Toronto Canada; Centre National de la Recherche Scientifique France; Peking University China

**Keywords:** absence, zero, perception, detection, numerosity, Human

## Abstract

The ability to represent absences is fundamental to human perception and cognition. Sensitivity to non-numerical absences depends on inferring a lack of sensory evidence, enabling the recognition of a lack of environmental resources or other objects. In turn, encoding numerical absence as a concept – in the form of the number zero – has enabled a multitude of technological and mathematical advances. Whether perceptual and conceptual absences rely on overlapping or distinct neural representations in the human brain, however, remains unknown. We employed three different tasks together with magnetoencephalography to characterise neural representations of absence evoked within perceptual and numerical domains. Using multivariate decoding and Bayes factor analyses, we replicate previous findings of shared representations of numerical absence across symbolic and non-symbolic formats. In contrast, we show that these representations of numerical absence are distinct from those evoked by non-numerical absences. This result held even after controlling for stimulus-level confounds which generate surface-level similarities between perceptual absences and non-symbolic empty sets. Our results extend a boundary between perception and cognition to the domain of absence and provide insight into the different mechanisms employed by the human brain to both perceive and conceive of absences.

## Introduction

When describing the central tenets of jazz, Miles Davis said: “It’s not the notes you play, it’s the notes you *don’t* play.” With this aphorism, he distilled a fundamental insight that perceptions of absence are core features of sensory experience. Just as we can recognise a friend in a crowded café, we can also notice their absence if they are late; just as we are aware of the birds calling outside our window, we can become aware of the silence once they stop. The perception of absence also has conceptual significance, indicating the lack of countable items in a set. Ultimately, the ability to represent absence as a symbolic, numerical concept underpins the emergence of the number zero in mathematics – a fundamental advance in human culture underpinning a range of technological and scientific advances (Kaplan, 1999).

How these two types of absence – perceptual and conceptual – are inter-related psychologically and neurally remains poorly understood. On the one hand, it has been hypothesised that the conceptual representation of zero is continuous with a more fundamental and primitive representation of perceptual absence (Barton, 2020; Nieder, 2016). This hypothesis stems from findings of delayed mastery of both perceptual (Coldren and Haaf, 2000; Sainsbury, 1971) and numerical (Wellman and Miller, 1986; Wynn and Chiang, 1998) absences across development, and observations that children appear to scaffold the zero-concept onto notions of nothingness or absence when learning zero’s place at the start of the number line (Bialystok and Codd, 2000; Krajcsi et al., 2021; Yu et al., 2026). In adults, behavioural performance is often impaired for tasks involving zero compared to positive numbers (Brysbaert, 1995; Kutter et al., 2023) and this is mirrored in perceptual absences, where reaction times are often longer for stimulus-absence than stimulus-presence decisions (Kellij et al., 2021; Mazor et al., 2020; Mazor et al., 2026; Meuwese et al., 2014). On the other hand, a tradition in perception science recognises a boundary between perception and cognition, whereby perceptual and conceptual representations are distinct (Block, 2023; Firestone and Scholl, 2016). Applying this framework to the case of absence, we might expect that inferring perceptual absences – such as recognising a lack of birdsong – may be achieved via using distinct mechanisms to those supporting a representation of conceptual (e.g. numerical) absences such as zero. However, beyond an initial period of perceptual scaffolding of numerical absences in childhood, it remains unclear whether the neural underpinnings of perceptual and numerical absence in the adult human brain are overlapping or distinct.

Non-numerical perceptual absences have been shown to rely on dedicated neural machinery that are – at least in part – distinct from that underlying detection of stimulus presence. For example, in non-human primates, individual neurons in the prefrontal cortex specifically fire prior to reporting an absence of experience in a perceptual detection task (Merten and Nieder, 2012). Neurons with similar characteristics have also been found in the nidopallium caudolaterale of carrion crows (Wagener and Nieder, 2024), suggesting the active encoding of absence may be an evolutionarily preserved function that relies upon higher-order or associative brain regions. In line with this, single neurons in the posterior parietal cortex of humans show stimulus-locked responses to the failed detection of targets (Pereira et al., 2021). More generally, a capacity to self-monitor the reliability of perceptual systems may be important for distinguishing between bona fide perceptual absences and lapses in attention (Mazor et al., 2026).

Neural representations of the number zero show similarly distinct properties. Like non-numerical perceptual absences, numerical absences are actively represented in higher-order brain regions of non-human primates (Okuyama et al., 2015; Ramirez-Cardenas et al., 2016) and crows (Kirschhock et al., 2021). Importantly, two different coding schemes seem to underwrite single-cell representation of zero. Some cells represent zero in a continuous manner, firing maximally for numerical absence and showing a graded decrease in activation as numerical magnitude increases (Okuyama et al., 2015; Ramirez-Cardenas et al., 2016). In contrast, other cells show an exclusive response profile, responding specifically to zero and being equally inactive for all other magnitudes (Okuyama et al., 2015; Ramirez-Cardenas et al., 2016), reminiscent of a categorical representation of ‘nothing’ (vs. ‘something’). In humans, single cells in the medial temporal lobe have also been shown to encode numerical absence in both symbolic (‘0’) and non-symbolic (empty set) formats, with empty sets engaging partially distinct representations to countable numbers of dots at the population level (Kutter et al., 2024). Finally, in a previous study using magnetoencephalography (MEG) in humans, we recently identified neural representations of zero within human posterior parietal cortex which exhibited overlap between empty sets (i.e. blank squares) and symbolic zero (Barnett and Fleming, 2024).

Finding that conceptualisation of zero overlaps with representations of sensory absence in a detection task would offer support to accounts that propose the evolutionary and developmental trajectory of zero concepts emerge from a more basic ability to track an absence of sensation (Nieder, 2016; Barton, 2020). On the other hand, finding that neural representations of non-numerical and numerical absences are distinct would support an extension of a boundary between perception and cognition into the domain of absence perception (Block, 2023; Firestone and Scholl, 2016). To decide between these possibilities, we recorded patterns of neural activation in both detection and numerical tasks using MEG. Participants either detected stimuli amongst noise or performed rapid calculations with dot patterns and numerical symbols. In each task, we extracted multivariate neural patterns that reliably tracked numerical and perceptual absence. We find that representations of numerical absence robustly generalise across symbolic and non-symbolic numerical formats, but do not generalise to perceptual absences. Together our findings provide evidence in favour of distinct neural representations of perceptual and numerical absence.

## Results

**Figure 1.**
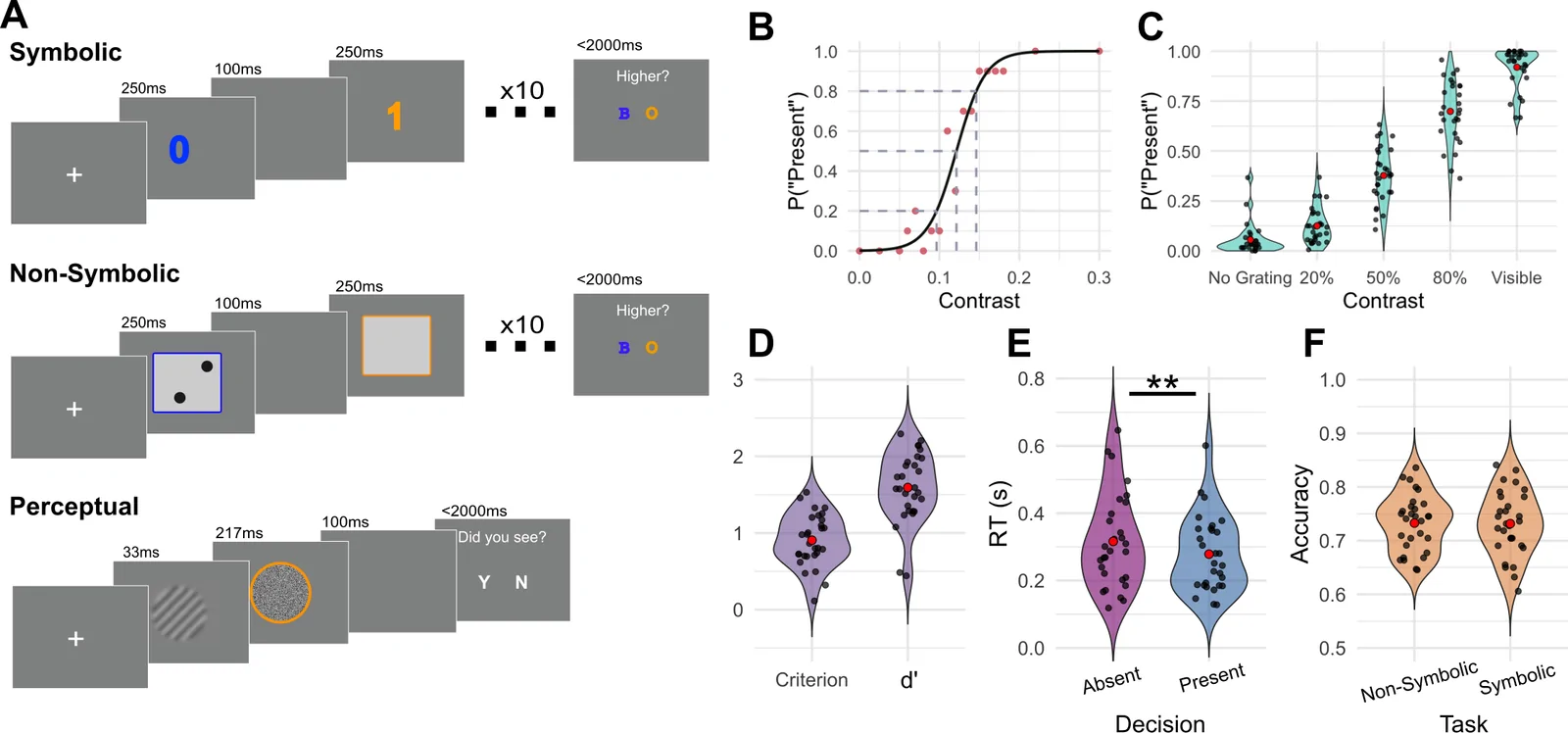
Tasks and behavioural performance. (**A**) Participants performed three tasks: two numerical (symbolic and non-symbolic) and one perceptual (detection). (**B**) Example participant’s psychometric function obtained during the calibration procedure. (**C**) The proportion of gratings reported as ‘present’ in the detection task. In all subplots (**C–F**), black dots are individual participants and red dots are the grand mean over all participants. (**D**) Criterion and d’ in the detection task. (**E**) Reaction times for absent and present responses in the detection task. (**F**) Accuracy in the symbolic and non-symbolic numerical tasks. ** denotes significance at p < .01.

Participants performed three tasks while undergoing MEG recording. They performed two numerical tasks which differed only in the format of numerical stimuli: non-symbolic dot patterns (empty sets to five dots) and symbolic numerals (‘0’ to ‘5’). In the numerical tasks, participants saw a rapid stream of 10 numbers, five of which were blue and five of which were orange (Figure 1A). At the end of the sequence, they reported whether the blue or orange set had a higher or lower average, counterbalanced across participants. In the perceptual task, participants were presented with a noisy grating at one of three calibrated SNR levels (Figure 1B and C). Participants were asked to report whether they saw the grating on each trial or not (Figure 1A). Using a range of SNR levels allowed us to gather an equal number of ‘present’ and ‘absent’ responses whilst controlling for stimulus contrast level (see ‘Decoding analyses’). Importantly, we used numerical and perceptual tasks that differed with respect to their structure and temporal dynamics. This ensured that any successful cross-decoding of absence could not be attributed to low-level or structural similarities between tasks, and instead would indicate a domain-general representation of absence. This approach builds on our previous work in which we identified a shared representation of numerical absence across non-symbolic and symbolic stimuli that generalised across task formats (Barnett and Fleming, 2024).

**Figure 1—figure supplement 1.**
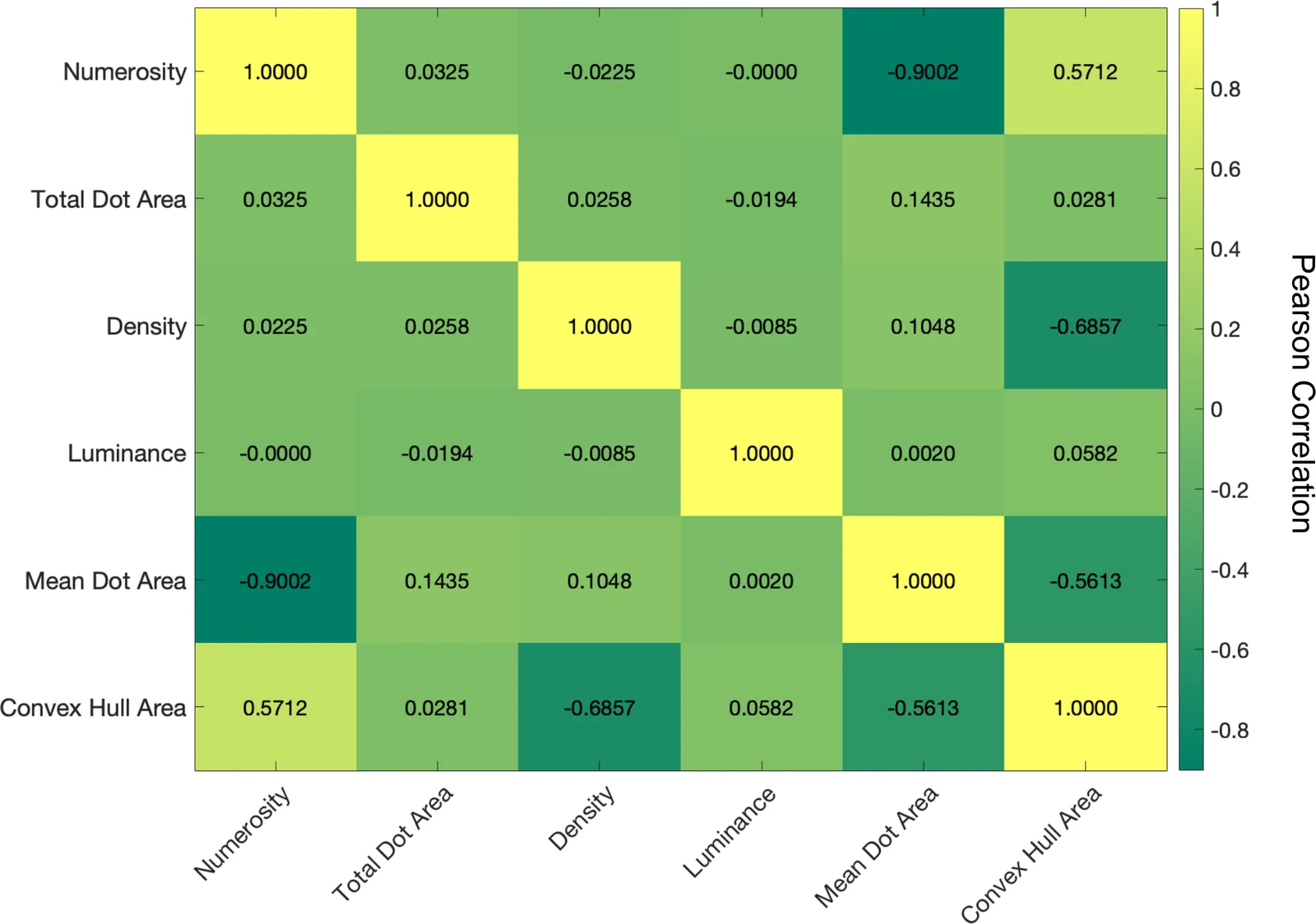
Visual features of non-symbolic dot stimuli. Total dot area was measured as the number of pixels covered by all dots. Density was computed as the negative median Euclidean distance between dots, such that higher distances between dots result in lower density scores. Luminance was computed using Weber contrast. Convex hull area was computed using the convhull and polyarea functions in MATLAB. Values are Pearson correlation (r) coefficients.

### Behaviour

In the perceptual detection task, participants were sensitive to the presence of the grating stimuli (mean d prime = 1.59, SD = 0.46, Figure 1D), while also showing a bias towards reporting the stimulus as absent (mean criterion = 0.91, SD = 0.34, Figure 1D). Replicating previous findings, participants took longer to report stimulus absence (mean RT = 0.32 s, SD = 0.14 s) compared to stimulus presence (mean RT = 0.28 s, SD = 0.11 s, t(28) = –3.0176, p=0.0054, Figure 1E). In the numerical tasks, participants were significantly above chance in reporting which stream had the higher average (symbolic: t(28) = 20.61, p<0.001; non-symbolic: t(28) = 23.53, p<0.001), and performance was similar between the symbolic and non-symbolic formats (mean symbolic accuracy = 0.73, SD = 0.06, mean non-symbolic accuracy = 0.73, SD = 0.5, t(28) = –0.121, p=0.90).

### Cross-format neural representations of numerical absence

**Figure 2.**
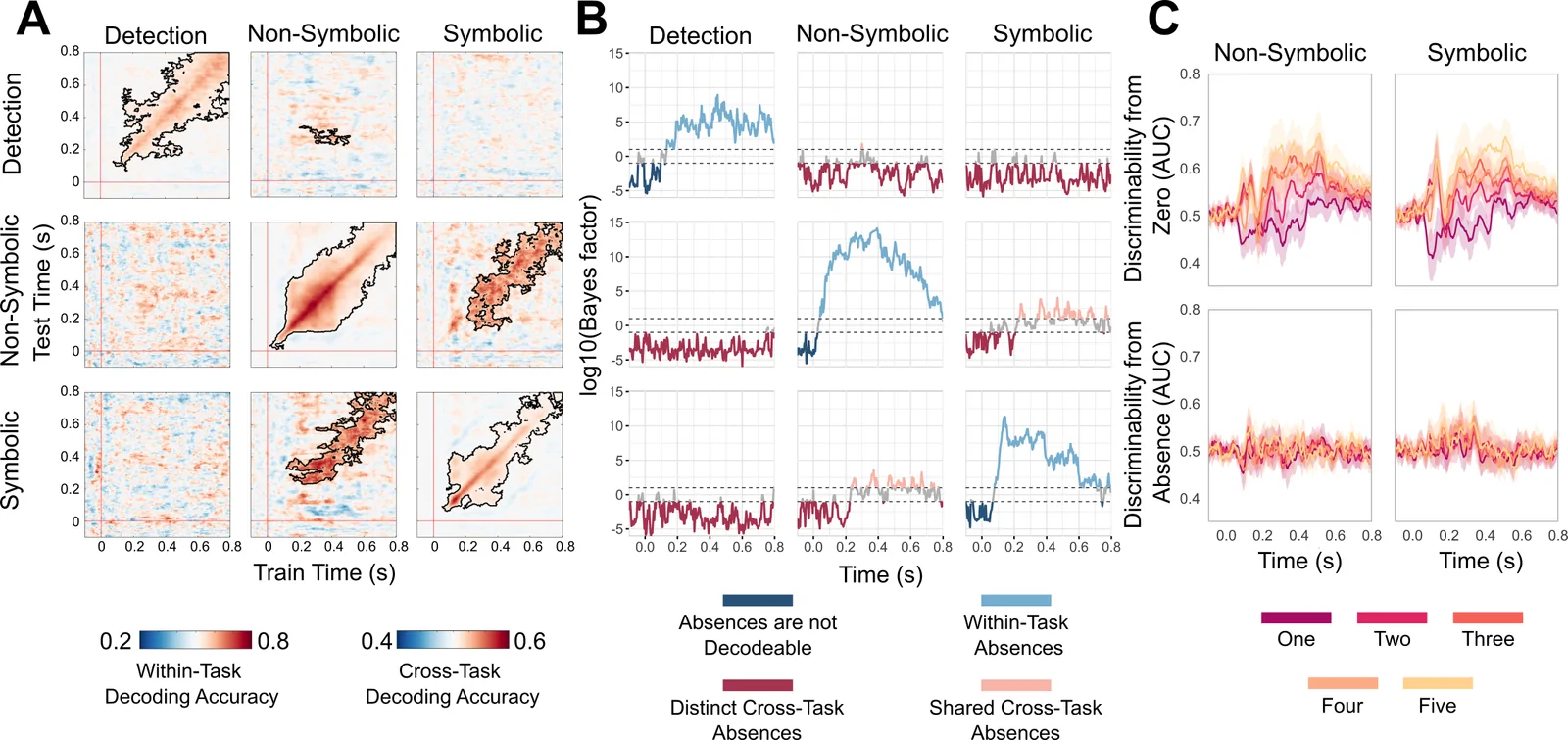
Distinct representations of perceptual and numerical absence. (**A**) Temporal generalisation decoding results. Representations of perceptual (top-left), non-symbolic (centre), and symbolic (bottom-right) absence were all decodable within each task, respectively. Numerical absences generalised across numerical formats (centre-right, bottom-centre) indicative of format-invariant representations of numerical zero. Representations of perceptual absence did not generalise to numerical tasks, except for a small cluster of significant decoding when the perceptual absence decoder was tested on non-symbolic empty sets (top-right). Black outlines represent areas of significant decoding as computed by cluster-based permutation tests. (**B**) Bayes factor analyses on diagonals of temporal generalisation matrices from (A). log10(Bayes factors) above 1 indicate strong evidence for above chance decoding and those below –1 indicate strong evidence for chance-level decoding. Black dotted lines indicate this threshold for strong evidence in both directions. There was decisive evidence for decoding of absences within each task (light blue). There was very strong evidence for shared representations of absence between symbolic and non-symbolic formats (pink). There was very strong evidence for distinct representations of perceptual and numerical absence (red). (**C**) Top: training a decoder to classify non-symbolic empty sets from non-symbolic numerosities and testing it on symbolic numbers in a one-vs.-all process revealed increasing discriminability as distance from zero increased (left). The same cross-format distance effect is observed when training a classifier on symbolic zero and testing it on non-symbolic numerosities (right). Bottom: there was no evidence for larger numerosities being more discriminable from perceptual absence than smaller numbers in either the non-symbolic (left) or symbolic (right) task. Shaded areas represent 95% CIs.

We first asked whether we could robustly identify neural activity patterns specific to numerical zero. We have shown previously using MEG that neural representations of numerical zero generalise between non-symbolic empty sets and symbolic zero (Barnett and Fleming, 2024). In line with this finding, binary decoders trained to discriminate zero vs. non-zero trials within each format revealed representations of zero that were decodable up to 800 ms post-stimulus (Figure 2A, diagonal; Figure 2B, diagonal). By testing these decoders in the alternative numerical format, we examined whether neural representations of zero in one format generalise to the alternative format. We observed successful generalisation from around 200–800 ms post-stimulus (train on non-symbolic: time-averaged log10(BF) [0.2 s, 0.8 s]=1.09; train on symbolic: time-averaged log10(BF) [0.2 s, 0.8 s]=0.74; Figure 2A and B), replicating our previous findings of shared representations of zero across symbolic and non-symbolic numerical formats (Barnett and Fleming, 2024).

**Figure 2—figure supplement 1.**
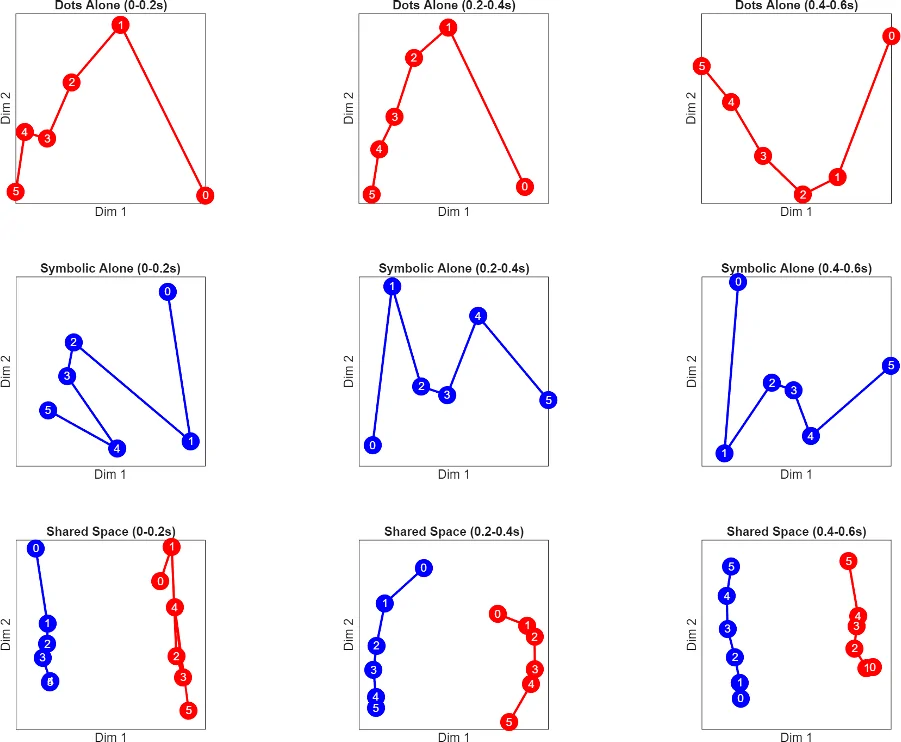
Multidimensional scaling of neural numerical representations. Multidimensional scaling reveals numerical representations to be ordered along a single axis (Dim. 1) almost immediately after stimulus onset (left column) through to 600 ms after stimulus onset (right column). Both symbolic numerals and non-symbolic numerosities are aligned along the same neural dimension (Dim 2, bottom row).

**Figure 2—figure supplement 2.**
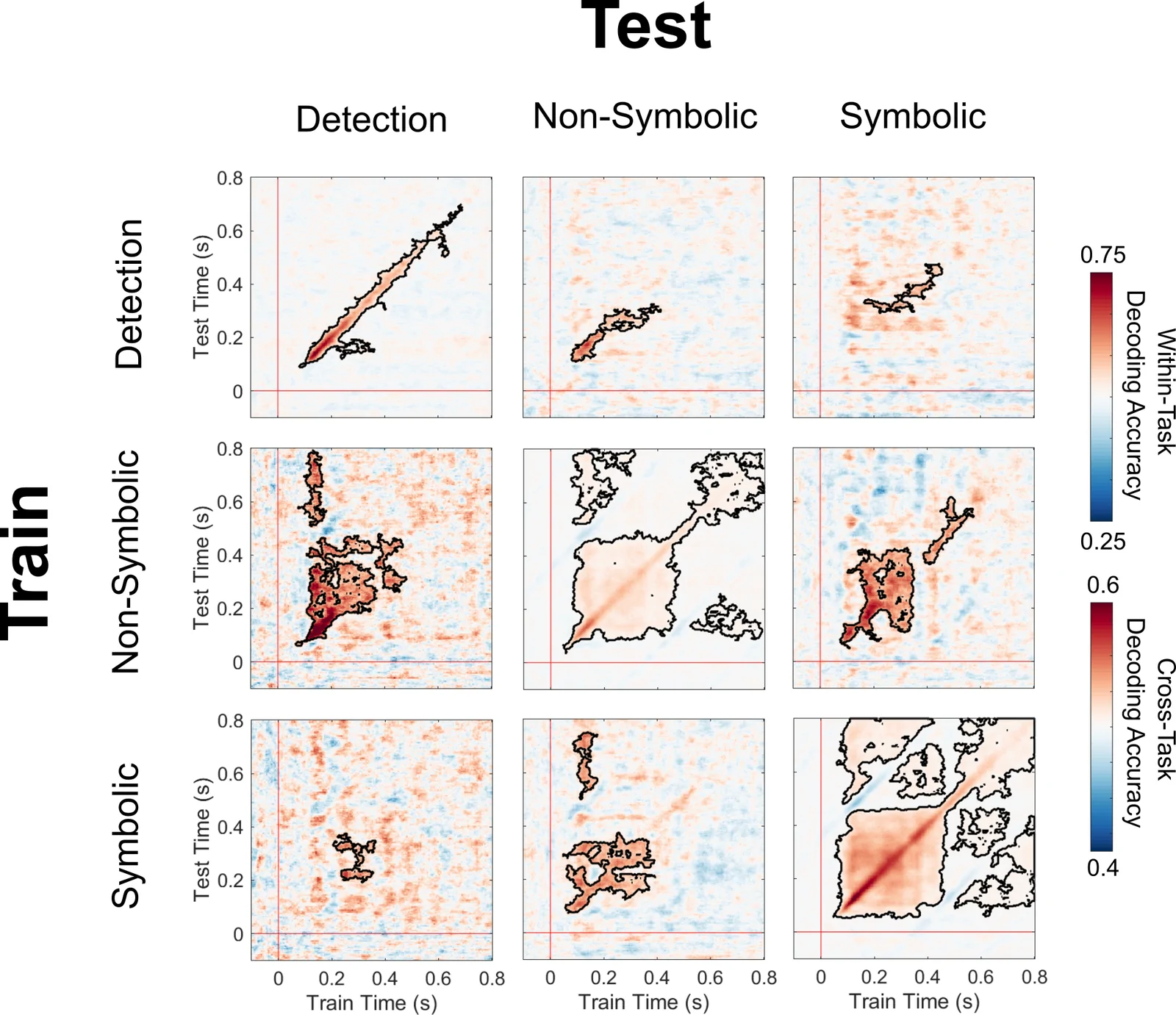
Colour can be decoded within and across all tasks. *Diagonal*: Within-task decoding of blue vs. orange stimuli was successful within all three tasks. *Off-diagonal*: Cross-task decoding of blue vs. orange stimuli was successful across all pairwise combinations of tasks and train-test direction. Clusters represent areas of significant decoding as computed by cluster-based permutation tests.

**Figure 2—figure supplement 3.**
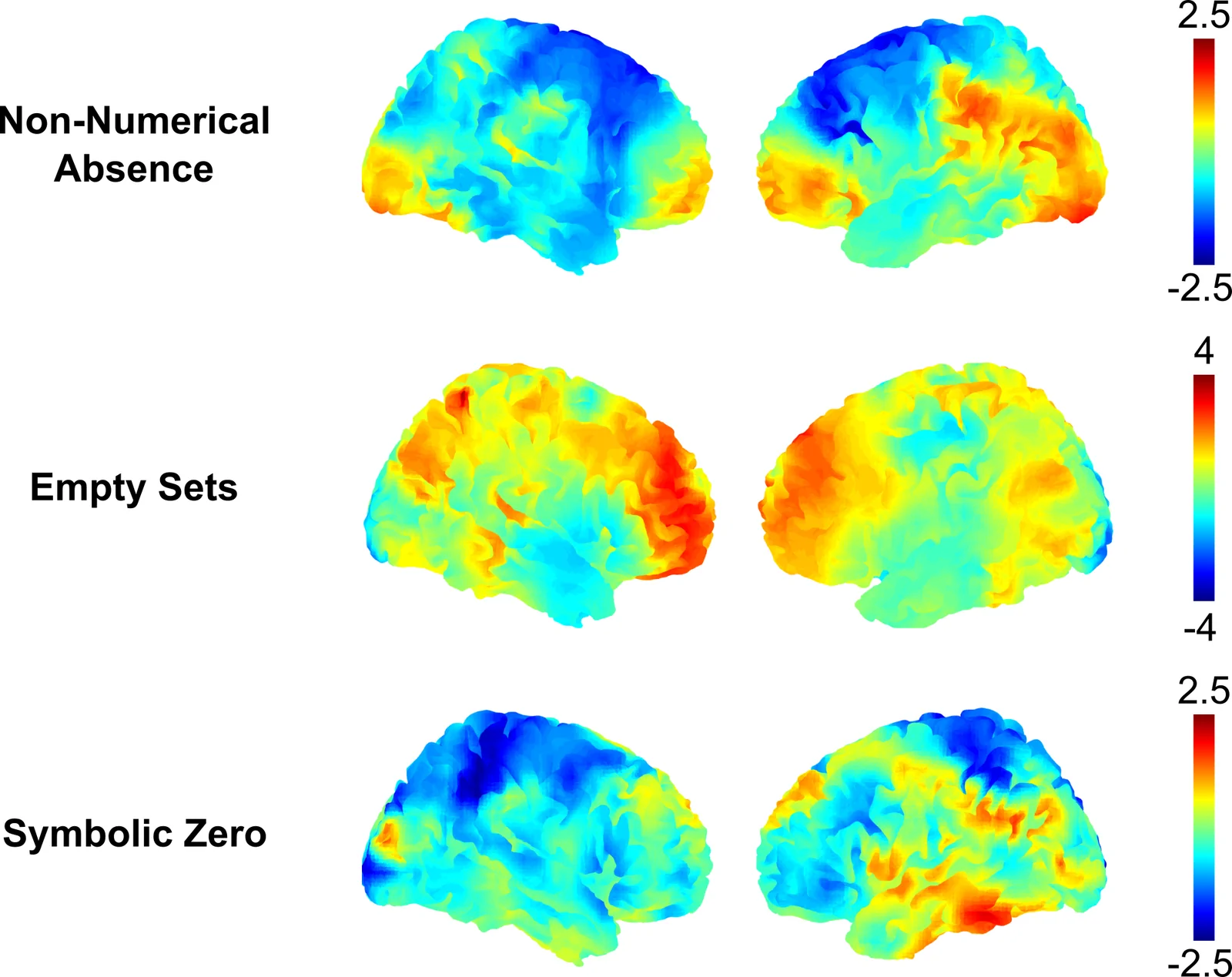
Source-level decoding weights for non-numerical absence, empty set, and symbolic zero decoders. Source reconstruction of decoding weights reveals the brain regions utilised by different decoders to classify different neural representations of absence. All decoding weights have undergone the appropriate transformation (Haufe et al., 2014), which in the case of a binary LDA is a contrast between the mean activity in each class. Colour bars represent the *t* statistic of this contrast. Maps are unthresholded for illustrative purposes.

**Figure 2—figure supplement 4.**
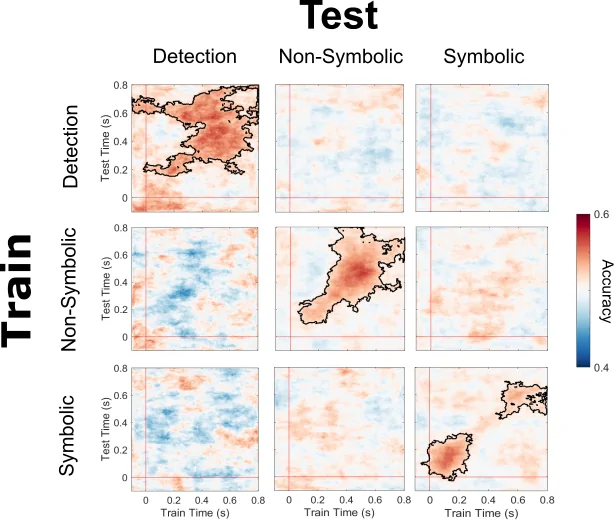
Representation of absence are domain-specific within the alpha band. Decoding the amplitude of the alpha rhythms identified representations of absence within all three tasks; however, none of these representations generalised across tasks. Clusters represent areas of significant decoding as computed by cluster-based permutation tests.

**Figure 2—figure supplement 5.**
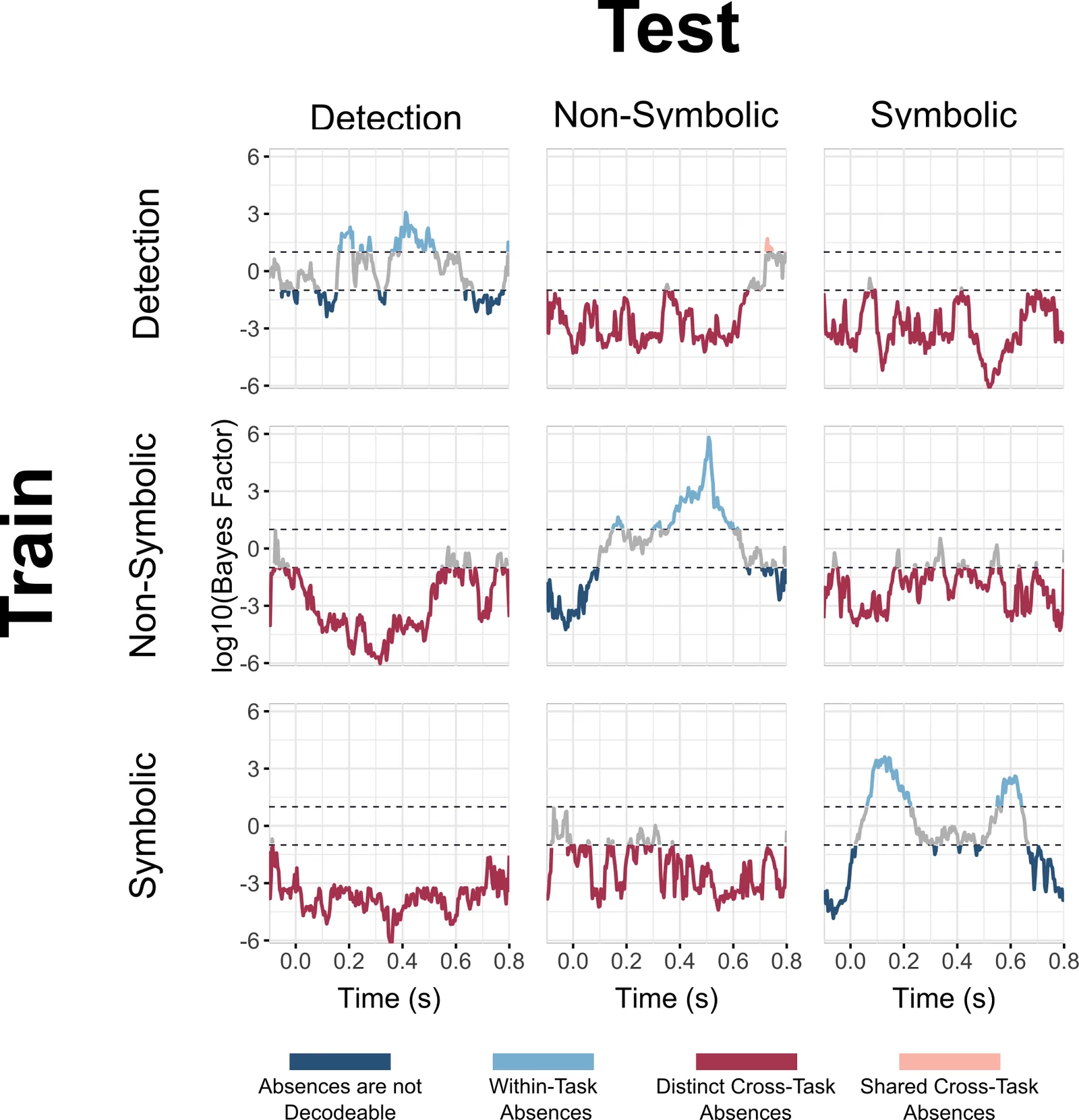
Bayes factors show strong evidence for domain-specific representations of absence in the alpha band. Bayes factors computed for the diagonal of the temporal generalisation matrices in Figure 2—figure supplement 4. There is strong evidence for domain-specific (and strong evidence against domain-general) representations of absence in the alpha band.

To determine whether these cross-format representations of zero exist on a graded neural number line, we used a one-vs.-one decoding approach to compute the discriminability between zero and each non-zero numerosity in the alternative numerical format. Neural representations induced by non-symbolic zero (empty sets) were more often confused with the symbolic numeral 1 or 2 than they were with symbolic numerals 4 or 5 (Figure 2C, top-left). Equally, neural representations of symbolic zero (‘‘0’’) were more often confused with one or two dots in the non-symbolic task, than they were with four or five dots (Figure 2C, top-right). Pairwise cluster-based permutation tests comparing the discriminability of different non-zero numerosities over time revealed clusters of significant differences in discriminability, where an increased distance from zero increased discriminability from cross-format zero (symbolic: 2 vs. 1, cluster p=0.002; 3 vs. 2, p=0.003; 4 vs. 3, p=0.15; 5 vs. 4, p=0.014; non-symbolic: 2 vs. 1, cluster p=0.001; 3 vs. 2, p=0.001; 4 vs. 3, p=0.27; 5 vs. 4, p=0.005). Multidimensional scaling of sensor-level data similarly revealed numerosities to be arranged along a single dimension encoding numerical magnitude that is shared between both numerical formats (Figure 2—figure supplement 1). These analyses confirm a graded representation of numerical absence that is evoked by both non-symbolic empty sets and symbolic zero (Barnett and Fleming, 2024), and establish that we are able to robustly recover neural representations of numerical absence in our paradigm using MEG.

### Neural representations of perceptual and numerical absence are distinct

We next asked whether representations of absence are shared between perceptual and numerical domains. We first trained a decoder to discriminate MEG signals corresponding to ‘absent’ vs. ‘present’ responses in the perceptual detection task, independent of stimulus contrast (see ‘Decoding analyses’). This allowed us to isolate neural representations underpinning inferences on perceptual absence while controlling for the influence of stimulus factors. Representations of perceptual absence were identified from 200 ms to 800 ms post-stimulus (Figure 2A, top-left), with Bayes factors (BFs) (Figure 2B, top-left) indicating strong evidence for successful decoding.

Having established successful decoding of both perceptual and numerical absences, we next asked whether the perceptual absence decoder could successfully discriminate zero from non-zero trials in the numerical tasks, and vice versa. This approach offers an empirical test of whether neural populations underpinning perceptual absence reports are also recruited when calculating with numerical zero. To allow us to obtain evidence for or against shared representations of numerical and perceptual absence, we computed BFs – with positive BFs indicating evidence in favour of generalised representations, and negative BFs indicating evidence in favour of distinct neural representations. To assist in visualisation, we plot log10 transformation of BFs such that BFs supporting either distinct or generalised representations are symmetric around zero. Interpretation of log10 BFs follows standard convention for interpreting BFs, with log10(BF) in the range [0,±0.5] offering weak evidence, [±0.5,±1] moderate evidence, [±1,±1.5] strong evidence, [±1,±2] very strong evidence, and [±2,±inf] being decisive (Jeffreys, 1998). To report log10 BFs in the text, we average BFs over the 0.2–0.8 s time window shown in Figure 2C to report a time-averaged log10 BF. In our plots, we indicate the time points when evidence for either hypothesis exceeds the threshold for strong evidence.

We note that within each domain (perceptual and numerical) there was strong evidence for decoding of absences, suggesting any failure to generalise between domains is not due to a lack of signal (Figure 2B, diagonal). We also included a control feature – stimulus colour (orange vs. blue) – that was common to each of the three tasks (Figure 1A). This feature showed both robust within-domain decoding, and, crucially, cross-domain generalisation for all pairwise combinations of tasks (Figure 2—figure supplement 2) – suggesting that any failure to observe cross-domain generalisation of perceptual/numerical absence is not due to a general failure to identify shared neural signatures across the different task contexts.

Overall, when training decoders on the detection task and testing on the numerical tasks, BFs indicated decisive evidence in favour of the hypothesis of distinct representations of perceptual and numerical absence throughout the trial epoch (Figure 2B). This was the case for both the symbolic (time-averaged log10(BF) [0.2 s, 0.8 s]=–2.91) and non-symbolic tasks (time-averaged log10(BF) [0.2 s, 0.8 s]=–2.16). Training decoders in the alternative direction (i.e. training on numerical tasks and testing on the detection task) also supported a hypothesis of distinct neural representations of perceptual and numerical absence (Figure 2B) (symbolic: time-averaged log10(BF) [0.2 s, 0.8 s]=–3.24; non-symbolic: time-averaged log10(BF) [0.2 s, 0.8 s]=–3.44). The only exception to this general pattern was when generalising from a non-symbolic empty set decoder to perceptual absences, which revealed a brief positive peak around 300 ms post-stimulus consistent with evidence for shared representations; however, the time-averaged BF across the full 200–800 ms window remained strongly negative, and in support of distinct representations (peak log10(BF)=1.83, time-averaged log10(BF) [0.2 s, 0.8 s]=–2.16) (Figure 2A, top-middle, Figure 2B, top-middle). Figure 2—figure supplement 3 illustrates linear discriminant analysis (LDA) decoding weights used for each of the three absence decoders in source space. We also established that pre-stimulus neural representations of perceptual and numerical absence identified in the alpha band were not shared between tasks (Figure 2—figure supplements 4 and 5).

If representations of absence are shared between perceptual and numerical domains, we would expect perceptual absences to show signatures of a neural number line, and be less discriminable from lower numerosities than higher numerosities. To test this, we again performed a one-vs.-one cross-decoding analysis, where we tested our perceptual absence decoder (absent vs. present decision) on different pairs of zero and non-zero numerosities (i.e. 0 vs. 1; 0 vs. 2; 0 vs. 3; 0 vs. 4; 0 vs. 5). Again, in line with distinct neural representations of perceptual and numerical absence, larger numerosities did not show a significant increase in discriminability from perceptual absence when compared to either smaller non-symbolic numerosities (all ps >0.157; Figure 2C, bottom-left) or symbolic numerals (all ps>0.130; Figure 2C, bottom-right).

### Visual features of perceptual absences generalise to empty set stimuli

Taken together, our analyses indicate strong evidence (as derived from BFs) that neural representations of perceptual and numerical absence can be robustly identified in patterns of MEG activity, and that these representations are distinct rather than shared. As noted above, the one exception to this pattern was a small but significant cluster of activity around 300 ms that indicated some shared representation of non-symbolic and perceptual absences (Figure 2A and B). We considered that this exception may have been driven by uncontrolled shared variance in lower-level visual features of our detection and empty-set stimuli. One possibility is that the spatial frequency profile of non-symbolic dot patterns covaries with numerosity and can drive early visual responses to non-symbolic stimuli in numerical tasks. This has been shown in previous work where the aggregate Fourier power of dot stimuli correlates with numerosity (Paul et al., 2022). Additionally, stimuli containing dots naturally possess higher contrast than blank empty-set stimuli. Crucially, similar visual properties may also characterise our detection stimuli, with spatial frequency and contrast profiles differing between grating stimuli and noise patches. It is possible that these, or other, stimulus-related visual confounds may contribute to the brief cluster of generalisation observed between perceptual and non-symbolic stimuli. By explicitly attempting to decode the physical presence or absence of the grating (which up until now was controlled for in our analysis) we aimed to further characterise this component of visually driven shared variance.

**Figure 3.**
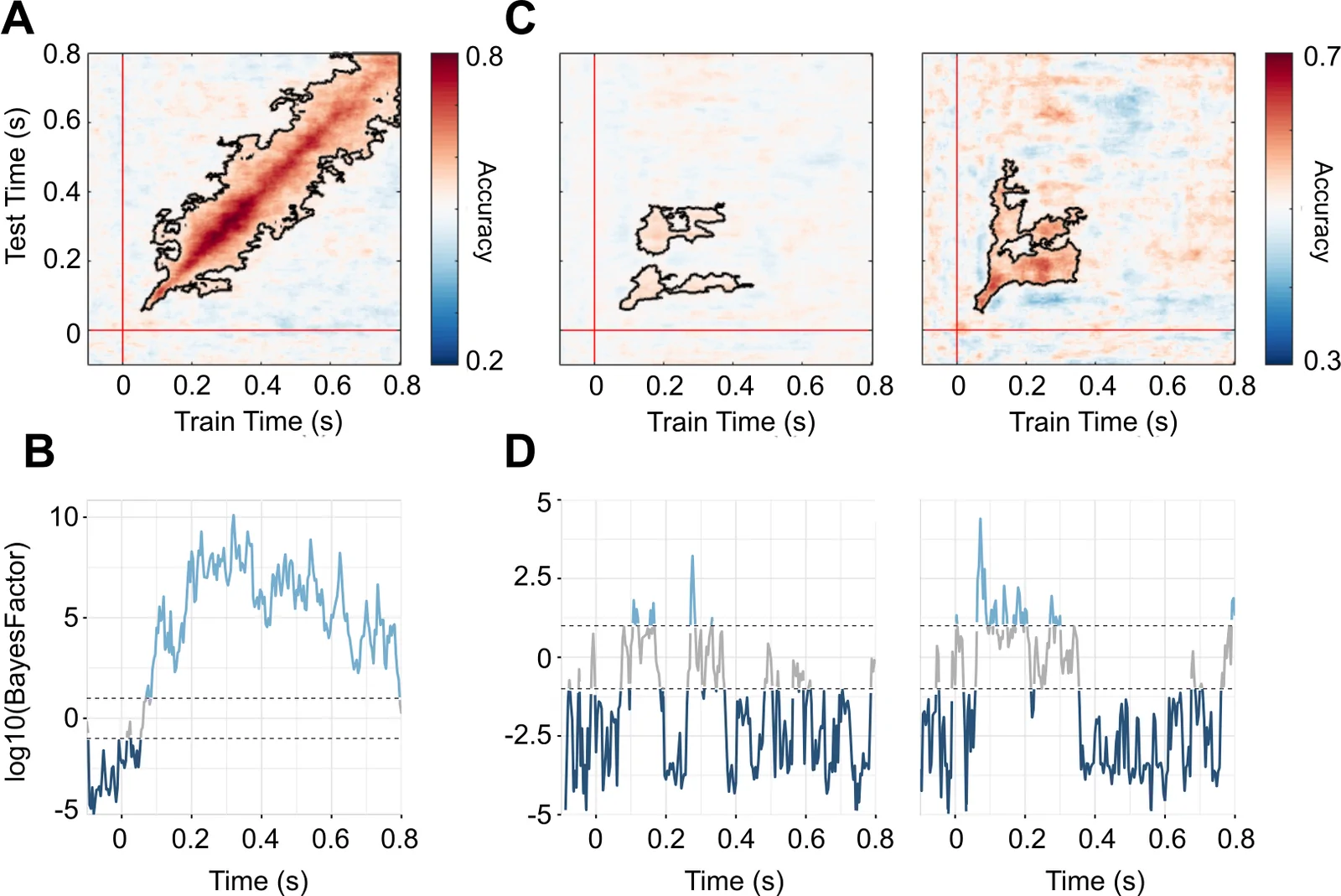
Stimulus features drive generalisation between detection and empty set stimuli. (A) A decoder trained to classify Hits vs. Correct Rejections could decode the presence and absence of grating stimuli from around 100 ms after stimulus onset. (B) Bayes factors exceeding the upper black dashed line (light blue) reflect strong evidence in favour of above-chance decoding. Bayes factors beneath the lower black dashed line (dark blue) reflect strong evidence in favour of chance-level decoding. (C) Cross-decoding between grating presence and absence (**A**) and empty set vs. non-zero dot stimuli. *Left:* Training on the detection stimuli and testing on non-symbolic stimuli. A cluster of significant generalisation was observed between 100 and 300 ms post stimulus onset. *Right*: Training on empty set vs. non-zero stimuli and testing on grating presence vs. absence in the detection task. Again, a cluster of significant cross-decoding was observed between 100 and 300 ms following stimulus onset. (D) *Left:* Bayes factors indicated moderate to strong evidence in favour of generalisation when testing on non-symbolic stimuli. *Right:* Bayes factors indicated strong evidence in favour of successful generalisation (light blue) when testing on perceptual presence vs. absence. Black outlines represent areas of significant decoding as computed by cluster-based permutation tests.

To evaluate this possibility, we trained a decoder to classify Hits vs. Correct Rejections within the detection task (Figure 3A). This decoder should be not only sensitive to the ‘present’ or ‘absent’ decision made by a participant, but also the physical presence (or absence) of a grating. To maximise sensitivity in this analysis, only trials within the supra-threshold contrast level were used to define Hits. We then tested this decoder on empty sets vs. non-zero dot patterns and found a cluster of significant generalisation between 100 ms and 300 ms, with the peak BF indicating evidence for shared neural signatures across the two tasks (peak log10(BF)=3.25; time-averaged log10(BF) [0.05 s, 0.3 s]=–0.82; Figure 3B, left). When decoding in the alternative direction (training on empty sets vs. non-zero dot patterns and testing on Hits vs. Correct Rejections), we observed similar patterns of generalisation, with evidence in favour of shared neural signatures between the two stimulus sets (peak log10(BF)=4.45; time-averaged log10(BF) [0.05 s, 0.3 s]=0.82; Figure 3B, right). Taken together, this result suggests that visual features (for instance, covarying spatial frequency profiles) may spuriously drive cross-decoding of absence in perceptual and non-symbolic numerical domains, unless stimulus contrast is appropriately controlled for. Indeed, when such properties are controlled for, generalisation across detection and non-symbolic tasks is almost entirely abolished (Figure 2). It is therefore all the more striking that, despite the potential for uncontrolled stimulus-driven contributions to cross-decoding, we find strong evidence against shared neural representations of perceptual and numerical absence (Figure 2).

## Discussion

Absences are fundamental to human consciousness and culture. Just as sensitivity to absences is pivotal for perception, the conceptualisation of absence as ‘zero’ enabled technological advances ranging from accounting to astronomy (Kaplan, 1999). Yet how these two kinds of absence – perceptual and conceptual – are encoded in the brain, and specifically the extent to which they rely on shared neural representations, has remained unknown. One proposal holds that the brain scaffolds conceptual representations of absence on top of more basic sensory representations of absence (Barton, 2020; Nieder, 2016). An alternative view holds that perceptual and conceptual absences are distinct and supported by separate neurocognitive resources (Block, 2023; Firestone and Scholl, 2016). Through a series of MEG decoding analyses, we provide evidence for the latter hypothesis, revealing distinct neural representations of absence in perceptual and numerical domains. Our findings extend previous proposals of a distinction between perception and cognition into the domain of absence perception and offer insight into the different mechanisms employed by the brain to both perceive and conceive of absences.

Whether perception and cognition can be demarcated according to the format of their representations is debated (Block, 2023; Quilty-Dunn, 2016; for review see Clarke and Beck, 2023). Classical distinctions describe perceptual representation as iconic, analogue, and non-propositional, while cognitive representations are thought to be conceptual and sentence-like (Block, 2023; Burge, 2018). Our data lend support to the thesis that conceptual and perceptual absences respect a boundary between perception and cognition. It is important to note, however, that the extent to which absences are in fact perceived remains debated. For instance, findings showing that absences do not ‘pop out’ in visual search tasks in the same way as presences (Treisman and Gormican, 1988) have been used to argue that absences are not represented in the visual system and that recognising an absence must always invoke some conceptualisation (Block, 2023). On the other hand, absences follow signatures of perceptual illusions (Goh et al., 2023; Phillips, 2013), with theoretical perspectives suggesting they are perceived either via inference (Mazor, 2025) or direct perception (Farennikova, 2013). Our results shape this debate by revealing that neural representations of non-numerical perceptual absence are qualitatively distinct from neural representations of both symbolic and non-symbolic numerical absences.

In order to interpret the lack of generalisation of absence representations between the detection and non-symbolic numerosity tasks, it is also useful to consider the representational status of non-symbolic numerosities. A large body of both behavioural (Burr et al., 2018; Burr and Ross, 2008; Cicchini et al., 2016) and neuroimaging (Castaldi et al., 2019; Park et al., 2016; Piazza et al., 2004) work has suggested that non-symbolic numerosities are represented as a primary perceptual attribute in the visual system, on a par with properties such as contrast, size, and orientation. In this light, a lack of absence generalisation between the detection and non-symbolic numerosity tasks would not delimit a boundary between perceptual and conceptual absences, but rather support a boundary between numerical and non-numerical perceptual absences. Such a divide may be due to perceptual-numerical absence representations requiring additional processes mapping non-symbolic empty sets onto a neural number line (Barnett and Fleming, 2024; Kutter et al., 2024), which is not required – and, as the present results seem to indicate, not instantiated – by non-numerical perceptual representations.

Perceptual and numerical absences are not the only kinds of absence represented in the brain. The capacity to know you don’t know – or are unable to remember – something plausibly depends on the capacity to represent absences at a metacognitive level. In non-human primates, it has been shown that metacognition about absences of mnemonic evidence (i.e. decisions about one’s own ignorance) rely on frontopolar cortex, while evaluating decisions about the presence of mnemonic evidence (i.e. decisions about the things one has seen) engages dorsolateral PFC (Miyamoto et al., 2018). One interpretation of this dissociation is that evaluating an absence of internal evidence requires an additional level of abstraction about one’s own cognitive states that is not required when evaluating evidence in favour. Consistent with this proposal, medial frontopolar cortical activation in humans preferentially tracks confidence about reports of stimulus absence relative to reports of stimulus presence (Mazor et al., 2020). One intriguing possibility, therefore, is that the metacognitive evaluation of different absence experiences is served by a general abstraction process that recruits the frontopolar cortex. Whether or not such a system is required for evaluating decisions involving numerical absence remains to be tested.

Evidence from developmental studies indicates that the process of learning a zero-concept involves a gradual mapping of numerical and verbal properties to notions of nothingness and absence (Nieder, 2016). For instance, even before children possess quantitative knowledge about zero’s place on the number line, they can appreciate its relationship to ‘no things’ (Wellman and Miller, 1986) and even denote zero by leaving paper blank (Bialystok and Codd, 2000). In a recent study, preschool children demonstrated the ability to associate the word zero with perceptual absence and nothingness, but failed when it came to appreciating the word ‘zero’ as a number, or as a number-word that might apply to a specific noun (e.g., there are *zero fish*) (Yu et al., 2026). Even after quantitative knowledge of empty sets is established, however, meta-knowledge about zero’s status as a number remains fragile (Krajcsi et al., 2021), illustrating the gradual emergence of a symbolic, numerical concept from a category of ‘nothing’.

If zero is learned by mapping numerical concepts onto experiences of perceptual absence, then why do we find distinct representations of absence in the two domains? One explanation may be that, to master the linguistic and conceptual basis of zero, numerical representations of absence must be sufficiently abstracted away from sensory absences to be incorporated in a neural number line, ultimately resulting in distinct representations in the two cases. It has been suggested that the description of non-occurrences with positive labels can aid processing and memory of absence experiences (Hearst, 1991). Labelling nothingness with the word ‘zero’ may therefore not only bring the concept into the realm of numbers but could also create a positive conceptual indicator for the presence of ‘something’, namely the presence of an absence. We anticipate that young children may exhibit stronger representational overlap between perceptual and numerical representations of absence during the development of conceptual understanding of zero.

The tasks we employed in this study were dissimilar by design. This offered a strict test of the hypothesis that absences share representational currency across perceptual and numerical domains, because successful cross-decoding could not be artificially inflated by low-level task similarities across domains (Barnett and Fleming, 2024). However, this approach also carries the risk that task dissimilarity reduced our sensitivity to shared representations of absence. We think this is unlikely for several reasons: first, we focus on stimulus-triggered responses, and within each task, we are able to robustly decode stimulus-triggered absence representations; second, in our previous work we obtained robust generalisation of absence representations even when tasks are distinct, a finding we now replicate here with more closely matched tasks (Barnett and Fleming, 2024); and finally, our control analyses of colour do indicate positive generalisation across task format. Future work could explore the relationship between absences across domains using factorial designs that vary both domain and task independently, thus exposing possible interactions between absence representations and task context.

Absences play a foundational role in human perception and culture. Here, we show that the adult human brain harbours distinct representations of perceptual and numerical absences. Despite representations of numerical zero generalising between non-symbolic empty sets and symbolic numerals, neither of these numerical representations generalised to representations of perceptual absence. This result held even when controlling for low-level stimulus confounds which we show can drive spurious generalisation between tasks. Taken together, our results reveal distinct neural substrates underpinning perceptual and conceptual absences and extend a boundary between perception and cognition to include cases of absences.

## Materials and methods

### Participants

Thirty participants (*M*_age_: 27.07 years, *SD*_age_: 9.11) took part in the MEG experiment at the Department for Imaging Neuroscience, University College London. One participant performed at chance level in both numerical tasks and was therefore excluded from analyses. All analyses were performed on the remaining sample of 29 participants. Informed consent was given before the experiment and ethical approval was granted by the Research Ethics Committee of University College London (#1825/005).

### Stimuli

The detection task involved participants detecting a grating patch embedded within Gaussian noise. Gratings were oriented 45° clockwise from vertical, contained 2 cycles per degree and subtended 3° of visual angle. The signal to noise ratio of the grating and Gaussian noise was adjusted to sample a range of values from each subject’s psychometric function (see ‘Detection calibration procedure’). The masks were noise patches that were randomly generated on each trial and contained either an orange or blue border.

Numerical dot stimuli were created using custom MATLAB 2021b (MathWorks) scripts that are stored at https://github.com/benjybarnett/PerceptualNumericalAbsence, Barnett, 2026 and consisted of different numbers of dots (from 0 to 5) on grey backgrounds. Total dot area was controlled for by systematically reducing the size of the dots as the number of dots increased, such that the total number of pixels included in a stimulus were constant across numerosities. Dot sizes were held constant within each individual stimulus display. In both stimulus sets, 50% of dots were black and 50% were white, such that the contrast of the dot patterns did not increase with numerosity (the increase in contrast generated by an increasing number of black dots was cancelled out by the increased number of white dots). Figure 1—figure supplement 1 reports the correlation between total dot area, density, luminance, convex hull area, average dot size, and numerosity in the non-symbolic stimulus set, highlighting how our stimulus-generating procedure successfully controlled for the association between certain low-level visual properties and numerosity. Empty set stimuli contained only a grey background in both stimulus sets. To ensure participants could not rely on low-level visual cues in identifying empty set stimuli, the background luminance was varied within and across stimulus sets and the background square size was randomly varied across all stimuli (between 150 and 250 pixels squared). Given that various low-level visual features naturally covary with one another, it is difficult to control for all features in one stimulus set. However, the empty set stimuli that are the focus of our analyses of absence remain unaffected by visual properties covarying with numerosities above zero.

### Experimental procedure

The tasks were presented to subjects using MATLAB (MathWorks) and the Psychophysics Toolbox (Brainard, 1997; Kleiner et al., 2007). Participants practiced the tasks on a computer before the MEG session. In the MEG scanner, participants first performed a calibration procedure for the detection task (see ‘Detection calibration procedure’). The tasks were then performed in alternating miniblocks of 60 detection trials, 22 symbolic trials, and 22 non-symbolic trials per MEG recording block. The order of the tasks was pseudo-randomly ordered within each block, and the starting order was counterbalanced across participants. There were 10 MEG blocks in total, resulting in 600 detection trials, 220 symbolic numeral trials, and 220 non-symbolic dot trials across the whole experiment. Participants responded using two buttons on a button box held in their right hand.

### Symbolic task

We modified the symbolic numeral averaging task introduced by Spitzer et al., 2017 to include the number zero (Barnett and Fleming, 2024). In one trial, 10 numerals ranging from 0 to 5 were presented in a random order (Figure 1A; *Symbolic Zero*). Five of the numerals were blue and five were orange. Each numeral was displayed for 250 ms with an interstimulus interval of 100 ms. The numerals were randomly selected on each trial to obey the constraint that the mean of the blue numerals could not equal the mean of the orange numerals. The response required at the end of each trial was counterbalanced across subjects, with half of the subjects reporting which set of numerals (orange or blue) had the highest average and the other half reporting which set had the lowest average. Participants had 2000 ms to respond, after which they were given feedback in the form of a green (correct) or red (incorrect) rectangle surrounding the response options. The button-response mappings were counterbalanced across blocks to prevent motor information contaminating our decoding analyses. Intertrial intervals were randomly sampled from a uniform distribution between 600 and 1000 ms.

### Non-symbolic task

The non-symbolic task followed the same procedure as the symbolic task (Figure 1A; *Non-symbolic*). The only difference was that, instead of seeing symbolic numerals, participants saw a stream of 10 dot patterns, five of which had an orange border and five had blue borders. The rest of the procedure was identical to the symbolic numeral task.

### Detection calibration procedure

When participants were set up in the scanner, they first performed a calibration block for the detection task. The trials followed the same protocol as the detection task. A grating was presented for 33 ms and was followed by a 217 ms backward mask consisting of Gaussian noise (Figure 1A; *Sensory Absence*). Following stimulus presentation, participants reported whether they saw the grating or not. A total of 17 SNR values were sampled from 2.5% to 30%, with 10 trials per SNR. 20 additional pure noise trials were included, where no grating was presented. This process allowed us to fit participants’ psychometric functions (Figure 1B), observing the SNR values where participants had the probability of detecting a stimulus 20%, 50%, and 80% of the time.

### Detection task

The detection task followed the same task structure as the calibration block (Figure 1A; *Sensory Absence*). Within each experimental block, 10% of trials were no-stimulus catch trials where no grating was presented. On another 10% of trials, we presented grating stimuli above threshold (30% SNR). The remaining 80% of trials were evenly split between SNR values computed from the calibration block (P(Present) = [20%, 50%, 80%]). Within each block, there were 16 trials per each SNR value (Figure 1C). The colour border to the backward masks was irrelevant to the task and participants were instructed to ignore the colours. Button response mappings were counterbalanced over blocks.

### MEG preprocessing

MEG data were recorded continuously at 600 Hz using a 273-channel axial gradiometer system (CTF Omega, VSM MedTech) while participants sat upright inside the scanner. To remove line noise, the raw MEG data were preprocessed with a Discrete Fourier Transform and bandstop filter at 50 Hz and its harmonics. A high pass filter of 0.5 Hz was also applied to the data.

MEG data were analysed using FieldTrip (Oostenveld et al., 2011) and MVPA-light (Treder, 2020). The numerical tasks were segmented into epochs of –500 ms to 4000 ms relative to trial onset. For the detection task, segments were specified from –400 ms to 1500 ms. Baseline correction was performed where, for each trial, activity in a pre-trial window (numerical: [–500, 0]ms, detection: [–200, 0]ms) was averaged and subtracted from the entire epoch per channel. Data were downsampled to 300 Hz. During artefact rejection, trials with high kurtosis were visually inspected and manually removed if they were judged to contain excessive artefacts. An independent components analysis was carried out on the MEG data, and the components with the highest correlation with eye-tracking data were discarded after visual inspection. Components showing topographic and temporal signatures typically associated with cardiac artefacts were also removed by eye. This procedure was performed separately for the three tasks. Finally, a second stage of epoching was performed. In the numerical task, trials were segmented into –100 ms to 800 ms epochs around the onset of each numeral. Trials were then baseline corrected again using the pre-stimulus window. In the detection task, trials were segmented into –100 ms to 800 ms around target onset.

### Decoding analyses

To test whether representations of absence were shared between conceptual and perceptual domains, we used a series of decoding analyses. First, to reveal the temporal profile of format-specific and format-invariant representations of numerical zero, we trained a binary LDA decoder to decode zero vs. non-zero symbols and numerosities (Figure 2A). This was conducted using a temporal generalisation approach whereby a classifier is trained on each time point and tested on all other time points (King and Dehaene, 2014), revealing how stable neural representations are over time.

Within-format decoding involved training and testing a classifier to identify numerosities on trials from one format (e.g. numerals or dots). In cross-format decoding, we trained the classifier on one format and tested it on the other (e.g. training on symbolic trials and testing on non-symbolic trials, and vice versa). Importantly, cross-format decoding allows us to empirically assess whether neural patterns associated with different numerosities share a common neural code across formats. For statistical inference on within-task decoding, we conducted fivefold cross-validation. Cross-validation is not required in cross-format decoding because the test data are never seen by the classifier during training, and thus there is no risk of overfitting. In both analyses, prior to decoding, the non-zero numerosities were balanced and five trials per numerosity were averaged. Next, zero and non-zero numerosities were balanced. The resulting ‘zero’ decoders were uniquely trained to identify neural representations of numerical zero in symbolic or non-symbolic format (Figure 2A). As such, when tested on the alternative format, any successful cross-decoding is evidence for format-invariant representations of zero.

Next, we used binary decoders to reveal whether abstract representations of numerical zero exist on a graded number line. Here, we trained the decoders to discriminate zero vs. all non-zero numerosities (one to five) separately and then tested these binary decoders on the corresponding numerosities in the opposite format (Figure 2B). This resulted in five different classifiers per format (Barnett and Fleming, 2024). This was done in both decoding directions: training on non-symbolic trials and testing on symbolic numerals; training on symbolic numerals and testing on non-symbolic trials. We used the area under the receiver operating characteristic as a metric for discriminability between each pair of classes. In line with the hypothesis that format-invariant representations of zero exist on a graded, abstract neural number line, we expected discriminability to improve as numerical distance from zero increased. To test this effect statistically, we performed one-tailed, paired comparisons between the discriminability of successive numbers with zero (e.g. by comparing 0–2 vs. 0–1, 0–3 vs. 0–2).

To examine whether representations of absence were shared across numerical and sensory domains, we trained a binary decoder to classify participants’ Hits (where a stimulus is presented and the participant reports ‘present’) vs. Misses (where a stimulus is presented and the participant reports ‘absent’) in the detection task (Figure 3A). Stimulus-absent and above-threshold trials were removed from this analysis. An equal number of Hits and Misses were used from each of the other three SNR values used in the experiment. As such, there was equivalent stimulus information across trials with ‘present’ and ‘absent’ responses, limiting the contribution of low-level visual features contributing to the decoding of neural correlates of perceptual detection. This decoder was then tested on the two numerical tasks, which again had been sorted into zero and non-zero trials (Figure 3B). Decoding was done in both directions (training on detection, testing on numerical tasks, and vice versa). Similar to the analysis examining whether cross-format representations of zero were graded, we took the Hits vs. Misses decoder and tested it on individual pairs of numbers (e.g. 0–1, 0–2) in both numerical formats (Figure 3C). If sensory absence shares representational properties with numerical zero, we would expect to see greater discriminability of larger numbers from sensory absence than smaller numbers. Again, to test whether this was the case, we performed one-tailed, paired comparisons between the discriminability of successive numbers with sensory absence (e.g. by comparing absence-2 vs. absence-1, absence-3 vs. absence-2).

Previous work has found neural activity in the alpha band to predict reports of stimulus presence and absence (Mathewson et al., 2009; Samaha et al., 2017). To extract alpha band activity from our data, we bandpass filtered our continuous, unepoched data between 8 and 12 Hz and performed a Hilbert transform. Taking the absolute value of the Hilbert transformed data returned the amplitude of the alpha band signal over time, which could then be used as input to our decoder in the same way as analyses described above used the broadband MEG signal. The same trials were removed from analysis as those identified in the initial visual artefact rejection procedure. The same cross-decoding analyses were then performed on the alpha amplitude data, testing decoders within each task (Hits vs. Misses; Empty Sets vs. Non-Zero Dots; Symbolic Zero vs. Non-Zero Numerals) and across tasks (Figure 2—figure supplements 4 and 5).

Finally, to test whether low-level visual features of empty set and grating stimuli drive similar neural activity, we trained a decoder to discriminate stimulus-absent trials from above-threshold trials (Correct Rejections vs. Hits) in the detection task and tested this on empty set and non-zero dot patterns (and vice versa) (Figure 4).

For all decoding analyses, we used binary LDA decoders in conjunction with the MVPA-light toolbox (Treder, 2020) integrated with FieldTrip. To improve the robustness of the classifier, we applied L1-regularisation to the covariance matrix, and the shrinkage parameter was automatically determined using the Ledoit–Wolf formula within each training fold (Ledoit and Wolf, 2004). All decoding hyperparameters were chosen to match those of our previous work on numerical absence decoding (Barnett and Fleming, 2024), such that results would be directly comparable across the two studies.

### Statistical inference

Cluster-based permutation testing was used to correct for multiple comparisons during statistical inference (Maris and Oostenveld, 2007). For all analyses, 1000 permutations were employed with a cluster-forming alpha parameter of 0.05 and a significance threshold of 0.05. It is important to emphasise that cluster-based permutation testing does not provide precise information about when neural representations emerge. This limitation arises because cluster-level statistics are only defined for broader time windows that naturally encompass multiple individual time points (Sassenhagen and Draschkow, 2019).

### Bayes factor analyses

Cluster-based permutation tests are frequentist and as such cannot provide evidence for the null hypothesis (Dienes, 2011). In our case, this means that absence of significant (defined by frequentist permutation tests) cross-decoding between detection and numerical tasks cannot be interpreted as evidence for distinct representations of absence. To remedy this, we computed BFs by applying Bayesian *t*-tests to decoding accuracy values across participants, using a Cauchy prior centred on zero with a scale parameter of 0.7071. For directional hypotheses, a half-Cauchy prior was specified with a lower bound of 0.5. This lower bound was specified to allow for empirical decoding accuracies slightly above theoretical chance level (i.e. within an effect size of 0.5) to still provide evidence for the null. This is because decoding of neuroimaging data can often result in empirical chance levels that differ from theoretical chance (Stelzer et al., 2013; Teichmann, 2022). A lower bound of 0.5 was selected because multiple MEG decoding datasets have exhibited effect sizes up to 0.5 in baseline windows, where chance-level decoding is expected (Teichmann, 2022). Analyses were performed on the diagonal of the temporal generalisation matrices, resulting in a time-resolved vector of BFs that could indicate evidence in support of either successful cross-decoding or an absence of cross-decoding. Our interpretation of BFs follows standard thresholds (Jeffreys, 1961) which we log-transformed for visualisation and interpretability. Importantly, because Bayesian analyses are not related to overall error rates and are not used to threshold significance, they do not require correction for multiple comparisons (Teichmann, 2022).

### Source reconstruction

Both FieldTrip’s template single shell head model and its standard volumetric grid (8 mm resolution) were warped to participants’ individual fiducial points, generating a subject-specific forward model aligned in MNI space. Source reconstruction was performed using a linearly constrained minimum variance (lcmv) beamformer which applies spatial filters to the MEG data to generate source-level time courses. To reduce the impact of noise on the source estimates, we used a regularisation parameter of lambda = 5%. For each task, spatial filters were calculated by combining the leadfield matrix with the data covariance matrix across all stimuli and the timepoints 100–450 ms. These spatial filters were then applied to absence trials and presence/non-zero trials separately, generating reconstructed maps of source activity for different trial types. The maps can then be contrasted to reveal the source-level decoding weights associated with binary LDA decoding (Haufe et al., 2014; Figure 2—figure supplement 3).

## Data Availability

MEG data has been deposited at https://osf.io/xhqfc/ and all code for the analyses presented in this paper are openly accessible at https://github.com/benjybarnett/PerceptualNumericalAbsence (copy archived at Barnett, 2026). The following dataset was generated: BarnettB 2026Perceptual and Numerical AbsenceOpen Science Framework10.17605/OSF.IO/XHQFC

## Additional files

MDAR checklist

## Appendix 1

### Alpha amplitudes encode domain-specific representations of absence

Pre-stimulus neural activity in the alpha band is known to predict reports of stimulus presence and absence (Mathewson et al., 2009; Samaha et al., 2017). As such, it is possible that domain-general representations of absence may be encoded within the alpha frequency and decoding of broadband signals may lack the sensitivity to reveal them. To remedy this, we extracted the amplitude of participants’ alpha rhythms over time and used this as input to decoding analyses. Training decoders to classify absence vs. presence and zero vs. non-zero within the detection and numerical tasks respectively was successful (Figure 2—figure supplement 4, diagonal), indicating the existence of domain-specific representations of absence encoded within the amplitude of the alpha band. However, when these decoders were tested on alternative domains, no above-chance decoding was observed (all ps>0.246; Figure 2—figure supplement 4, off-diagonal). Moreover, Bayes factors indicated strong evidence against domain-general representations of absence in the alpha band (Figure 2—figure supplement 5). Interestingly, this result held even between the two numerical tasks, suggesting format-invariant representations of numerical zero (Figure 2) are not underpinned by fluctuations in alpha amplitude.
