# Supplementary material for: Distinct neural representations of perceptual and numerical absence in the human brain
Source: eLife. 2026 Nov 5;15:RP112088. doi: 10.7554/eLife.112088 (PMC13739276; doi:10.7554/eLife.112088)
Supplement: Peer review history

# Editor report: eLife Assessment

**DOI:** 10.7554/eLife.112088.3.sa0

**Authors:** Nathan Faivre

**Affiliations:** Centre National de la Recherche Scientifique France

**Keywords:** Convincing, Important

This **important** study presents evidence that the human brain encodes perceptual absence and numerical absence through distinct neural codes, while confirming that symbolic and non-symbolic forms of zero share a common representation. The evidence supporting this dissociation is **convincing**, although the tasks differ substantially, and it remains unclear whether the result reflects a divide between numerical and non-numerical absence or between perception and abstract concepts. The work will be of interest to neuroscientists and psychologists studying numerical cognition and the boundary between perception and higher-level cognition.

# Referee report: Reviewer #1 (Public review)

**DOI:** 10.7554/eLife.112088.3.sa1

Summary:

The authors investigate whether the brain uses the same neural representations for "absence" when it comes to seeing nothing versus thinking of zero. To do this, they recorded MEG while participants performed two types of tasks: (1) a perceptual detection task where subjects reported the presence or absence of a faint visual stimulus, and (2) numerical comparison tasks where subjects saw streams of numbers or dot patterns (both including "0" or empty sets) and decided which of two color-coded streams had a larger average. Multivariate decoders were trained to distinguish "present" vs "absent" in the detection task and "zero" vs "non-zero" in the numerical tasks. As a sanity check, the authors first replicate that symbolic (digit "0") and non-symbolic (empty dot sets) zeros share a common neural code (cross-format generalization). Crucially, they find that this numerical "zero" code does not overlap with the code for perceptual absence: cross-decoding between the detection task and number tasks yields Bayes factors strongly favoring distinct representations. In other words, the brain's pattern for "no grating was seen" cannot decode the pattern for "the number zero was shown," and vice versa. A small brief cross-decoding effect around 300 ms was observed, which the authors attribute to low-level visual confounds (and which they test with an additional control decoder for stimulus presence), but overall, the evidence supports a dissociation.

Comments on revised version.

Thank you for the detailed response to my comments. The authors have adequately addressed my concerns, and I have no further comments at this stage.

# Referee report: Reviewer #2 (Public review)

**DOI:** 10.7554/eLife.112088.3.sa2

The authors tackle the question of whether conceptual absence is neurally encoded in the same way as perceptual absence. On one hand, the neural bases of perceptual absence have been largely investigated, as exemplified by the study of neural correlates of perception of aware vs. unaware stimuli, and on the other hand, the overlapping neural encoding of symbolic ('0') and non-symbolic (number of dots) formats of numerical absence has been previously established (Barnett & Fleming, 2024). However, the direct comparison of neural representations between numerical and perceptual absences remained to be investigated.

This article fills this gap by designing a Magneto-EncephaloGraphy (MEG) study using multi-voxel pattern analysis (MVPA) and temporal generalization to probe the similarity of neural patterns across the representation of perceptual absence (lack of stimuli), symbolic ('0'), and non-symbolic (number of dots) formats of numerical absence. They confirmed previously obtained evidence for shared neural representation across both formats of numerical absence. They report evidence for an absence of shared representation between both formats of numerical absence on one hand and perceptual absence on the other hand, while controlling for the confounding effect of low-level visual features. Their results overall support the conclusion that conceptual and perceptual absence are neurally encoded in a distinct way and speak in favour of a boundary between the representation of the concepts and the percept of absence.

Major strengths:

(1) Behavioral and neuroimaging results convincingly demonstrate that neural encoding of symbolic and non-symbolic absences is shared and situated on a graded, abstract neural number line, replicating previous results, notably from the authors themselves (Barnett & Fleming, 2024).

(2) They show that neural encoding of perceptual and numerical absence do not generalise across each other, while controlling for spurious confounds due to visual stimuli that are commonly shared in the cases of non-symbolic numerical absence and perceptual absences.

(3) They adequately use Bayesian analysis to distinguish absence of evidence vs. evidence of absence to support their claim.

(4) The interpretation of numerical absence as a representation of the concept of "nothingness" is adequate, although it might be further discussed by distinguishing the concept of "zero" on a number line from the concept of nothingness and that of an empty set (Nieder, 2016).

(5) The discussion about development and metacognition paves an interesting road for further investigation on the acquisition of the concept of zero, especially in light of debates on the progressive development of metacognitive abilities in children (Goupil & Kouider, 2019).

(6) Data and code are published with open-source access, allowing the community to further investigate the points as major weaknesses evoked below, if desired.

Comments on revised version.

I appreciated the authors' responses as they provided important additions to their discussion and supplementary information.

# Author comment: Author response

**DOI:** 10.7554/eLife.112088.3.sa3

**Authors:** Benjy Barnett, Stephen M Fleming

**Affiliations:** University College London London United Kingdom; University College London London United Kingdom

The following is the authors’ response to the previous reviews.

**Public Reviews:**

**Reviewer #1 (Public review):**

Summary:

The authors investigate whether the brain uses the same neural representations for "absence" when it comes to seeing nothing versus thinking of zero. To do this, they recorded MEG while participants performed two types of tasks: (1) a perceptual detection task where subjects reported the presence or absence of a faint visual stimulus, and (2) numerical comparison tasks where subjects saw streams of numbers or dot patterns (both including "0" or empty sets) and decided which of two color-coded streams had a larger average. Multivariate decoders were trained to distinguish "present" vs "absent" in the detection task and "zero" vs "non-zero" in the numerical tasks. As a sanity check, the authors first replicate that symbolic (digit "0") and non-symbolic (empty dot sets) zeros share a common neural code (cross-format generalization). Crucially, they find that this numerical "zero" code does not overlap with the code for perceptual absence: cross-decoding between the detection task and number tasks yields Bayes factors strongly favoring distinct representations. In other words, the brain's pattern for "no grating was seen" cannot decode the pattern for "the number zero was shown," and vice versa. A small brief cross-decoding effect around 300 ms was observed, which the authors attribute to low-level visual confounds (and which they test with an additional control decoder for stimulus presence), but overall the evidence supports a dissociation.

Strengths:

The authors investigate whether the brain uses the same neural representations for "absence" when it comes to seeing nothing versus thinking of zero. To do this, they recorded MEG while participants performed two types of tasks: (1) a perceptual detection task where subjects reported the presence or absence of a faint visual stimulus, and (2) numerical comparison tasks where subjects saw streams of numbers or dot patterns (both including "0" or empty sets) and decided which of two color-coded streams had a larger average. Multivariate decoders were trained to distinguish "present" vs "absent" in the detection task and "zero" vs "non-zero" in the numerical tasks. As a sanity check, the authors first replicate that symbolic (digit "0") and non-symbolic (empty dot sets) zeros share a common neural code (cross-format generalization). Crucially, they find that this numerical "zero" code does not overlap with the code for perceptual absence: cross-decoding between the detection task and number tasks yields Bayes factors strongly favoring distinct representations. In other words, the brain's pattern for "no grating was seen" cannot decode the pattern for "the number zero was shown," and vice versa. A small brief cross-decoding effect around 300 ms was observed, which the authors attribute to low-level visual confounds (and which they test with an additional control decoder for stimulus presence), but overall the evidence supports a dissociation.

We thank the reviewer for their careful reading of our work and their helpful comments and suggestions. Below, we respond to each of the reviewer’s concerns and highlight the relevant changes we have made in accordance with their suggestions.

Weaknesses:

R1C1. My main concern is whether the perceptual and numerical tasks are truly matched aside from their "absence" content. The perceptual task is a simple yes/no detection of a faint grating, whereas the numerical tasks involve holding two streams of 5 items in working memory and comparing their average. These tasks differ in many ways (stimulus complexity, decision rule, cognitive load), so it is possible that the lack of cross-decoding is due to general task differences rather than a fundamental "absence vs zero" dissociation. The authors do partially address this by showing that other shared aspects (like color) can cross-generalize, but one might still worry that an "absence" decision in a detection task engages different attentional or decisional mechanisms than a "zero" decision in a numerical context.

We thank the reviewer for prompting us to expand on this aspect of the design. We in fact deliberately chose to examine distinct perceptual and numerical tasks because we wanted to create a strict test of whether absence representations generalise. Having two closely-matched tasks would have led to a converse difficulty in interpreting any successful cross-decoding, had it been obtained. This is because it would have been difficult to ascertain whether cross-stimulus generalisation was because of a true domain-general representation of absence, or whether it was driven by other features of task design and stimulus-response contingencies. We employed the same strategy (of employing different tasks) in our previous work to identify cross-format representations numerical zero (Barnett & Fleming, 2024, Current Biology). Indeed, we think this was one of that study’s strengths, since the generalisation of ‘zero representations’ across numerical formats could not be explained by task similarity.

We appreciate though that our null findings shift the burden of proof to ensuring that this design choice has not resulted in a type 2 error. We think this is unlikely for several reasons: first, we focus on stimulus-triggered responses, and within each task, we are able to robustly decode stimulus-triggered absence; second, in our previous work we obtained robust generalisation of absence representations even when tasks are distinct, a finding we now replicate here with more closely matched tasks; and finally, as the reviewer indicates, our control analyses of colour do show positive generalisation across task format.

To make the motivation behind this design choice clearer to the reader, we have modified the following passage (p. 6):

Importantly, we used numerical and perceptual tasks that differed with respect to their structure and temporal dynamics. This ensured that any successful cross-decoding of absence could not be attributed to low-level or structural similarities between tasks, and instead would indicate a domain-general representation of absence. This approach builds on our previous work in which we identified a shared representation of numerical absence across non-symbolic and symbolic stimuli that generalised across task formats (Barnett & Fleming, 2024).

However, the reviewer is correct that this approach does not allow us to test whether unmeasured variables (such as cognitive load, decision making, etc) could interfere with cross-decoding, thus eliciting false negatives in our analyses. To elaborate on this limitation, and to suggest possible extensions to this work that could begin to help resolve this issue, we have added the following passage to the discussion (p. 19):

The tasks we employed in this study were dissimilar by design. This offered a strict test of the hypothesis that absences share representational currency across perceptual and numerical domains, because successful cross-decoding could not be artificially inflated by low-level task similarities across domains (Barnett & Fleming, 2024). However, this approach also carries the risk that task dissimilarity reduced our sensitivity to shared representations of absence. We think this is unlikely for several reasons: first, we focus on stimulus-triggered responses, and within each task, we are able to robustly decode stimulus-triggered absence representations; second, in our previous work we obtained robust generalisation of absence representations even when tasks are distinct, a finding we now replicate here with more closely matched tasks (Barnett & Fleming, 2024); and finally, our control analyses of colour do indicate positive generalisation across task format. Future work could explore the relationship between absences across domains using factorial designs that vary both domain and task independently, thus exposing possible interactions between absence representations and task context.

R1C2. The numerical averaging task closely resembles that used by Spitzer et al. (2017), who reported that both behavioral weighting and neural representational geometry exhibit anti-compression, with disproportionately stronger representations for larger numerosities. In contrast, the present manuscript interprets its decoding results as reflecting an ordered numerical continuum. It is therefore unclear whether the current analyses are sensitive only to ordinal structure or whether they also preserve the nonlinear representational geometry reported previously. This distinction is important because the interpretation of zero as part of a numerical continuum depends on the geometry of that continuum. The authors should clarify whether their representational analyses are compatible with the anti-compressed neural number line described by Spitzer et al., or explain why the two studies yield different conclusions.

We thank the reviewer for raising this interesting point. To address this question, we fitted the same model from Spitzer et al. (2017) to both numerical tasks in our data. The model fits two free parameters of interest: b, which controls the disproportionate bias to larger numbers and k, which controls the anti-compression (overweighting of extreme values) mentioned by the reviewer.

We found no difference in k values across any combination of tasks (Spitzer et al (2017) vs our non-Symbolic task; Spitzer et al (2017) vs our symbolic task; our non-symbolic vs symbolic task (all ps > .74)). The b parameter also did not differ significantly between any of the tasks (all ps > .06). Author response image 1 plots the decision values for each of the three tasks, illustrating the comparable weighting of extreme values and a consistent tendency to overweight larger values.

**Author response image 1.** Model fits to data from Spitzer et al. , (2017) and our two numerical tasks. Parameter values were consistent across all three tasks, replicating Spitzer et al.’s original finding of an anti-compressed number line with a bias towards larger numbers in both of our tasks (non-symbolic and symbolic) that extend the number line to include zero.

Seeing as our focus in the main paper is specifically on the representation of absence, we would prefer to retain these analyses in the publicly viewable response to reviewers rather than in the manuscript itself.

R1C3. The authors attempt to account for non-numerical visual information by controlling for Total Dot Area and Density. While this is an important control, these two variables do not exhaust the visual dimensions that covary with numerosity in dot displays. A large body of work has demonstrated that multiple continuous features, including average item area, total surface area, convex hull (field area), and density, are inherently intercorrelated and cannot all be independently controlled simultaneously (e.g., Piazza et al., 2004; Gebuis & Reynvoet, 2004; Castaldi et al., 2019; Karami et al., 2025). Consequently, controlling only two features does not fully establish that the decoded signal specifically reflects numerosity. To better characterize the stimulus space, I encourage the authors to report the correlation matrix among the principal visual features of the dot arrays (average item area, total surface area, convex hull/field area, density, and numerosity). In addition, it would be informative to quantify the unique contribution of each feature to the neural data using a multiple-regression RSA or semi-partial correlations RSA, similar to the analyses employed by Castaldi et al. (2019) and more recently by Karami et al. (2025). Such analyses would provide a more rigorous assessment of whether the decoded representations uniquely reflect numerosity after accounting for correlated visual properties.

We thank the reviewer for raising this point. We agree that a range of low-level visual properties of non-symbolic dot stimuli may covary with numerosity, and that it’s important to consider whether these properties may affect our results. As the reviewer points out, our original stimulus set was constructed with the aim of ensuring numerosity was uncorrelated with total dot area and dot density. We have followed the reviewer’s suggestion and now expand our correlation matrix to include not only the total dot area and density of dots, but also the convex hull and average dot area. We note that the empty set stimuli that are the focus of our analyses of absence remain unaffected by visual properties covarying with numerosities above zero.

To highlight these new analyses we have added the following sentence to our methods (p. 22):

“Numerical dot stimuli were created using custom MATLAB 2021b (Mathworks) scripts and consisted of different numbers of dots (from zero to five) on grey backgrounds. Total dot area was controlled for by systematically reducing the size of the dots as the number of dots increased, such that the total number of pixels included in a stimulus were constant across numerosities. In both stimulus sets, 50% of dots were black and 50% were white, such that the contrast of the dot patterns did not increase with numerosity (the increase in contrast generated by an increasing number of black dots was cancelled out by the increased number of white dots). Figure S1 reports the correlation between total dot area, density, luminance, convex hull area, average dot size, and numerosity in the non-symbolic stimulus set, highlighting how our stimulus-generating procedure successfully controlled for the association between certain low-level visual properties and numerosity. Given that various low-level visual features naturally covary with one another, it is difficult to control for all features in one stimulus set. However, the empty set stimuli that are the focus of our analyses of absence remain unaffected by visual properties covarying with numerosities above zero.”

The reviewer also suggests we perform RSA analyses to correlate visual features of our non-symbolic dot stimuli with the neural data to quantify the extent to which low-level visual features of the non-symbolic stimuli may be driving the ordered representations we observe in this task. While this analysis may help contextualise our findings on numerosities above zero, we think it is less relevant for understanding the degree to which absence representations are shared between numerical and perceptual domains. Indeed, for the empty set stimuli denoting absences in the non-symbolic task, many of the low-level visual properties discussed above cannot be computed. Here, instead, the primary visual confound of interest is that of similarities between empty sets and noise patches in the detection task, which we now tackle in response to the reviewer’s comment R1C6 below.

R1C4. The manuscript reports predominantly diagonal temporal generalization for non-symbolic numerosity, implying a rapidly evolving neural code. However, a recent study using time-resolved decoding of numerical representations (Karami et al., 2025) reported substantial off-diagonal temporal generalization, consistent with a temporally stable representational format. Although methodological differences between the studies may account for this discrepancy, the apparent contrast deserves discussion. In particular, it would be useful for the authors to clarify whether the differences arise from task demands, stimulus characteristics, preprocessing and decoding procedures, or from theoretical differences in what is being decoded. More generally, these findings raise the possibility that the temporal stability of numerical representations is task-dependent rather than fixed. If so, it would be interesting to discuss whether task demands might also influence the relationship between perceptual and conceptual representations of absence. Such a possibility could help explain why cross-decoding was not observed in the present study and suggests an interesting direction for future research.

We thank the reviewer for prompting us to consider differences in our results and those of Karami et al (2025). The reviewer is correct that, in the present manuscript, our decoding exhibits a diagonal pattern. However, the critical difference between these analyses and those in Karami et al (2025) are that we are decoding across numerical format and perceptual domain, while Karami et al decode within a single non-symbolic format. Indeed, in our previous work (Figure 2B, Barnett and Fleming, 2024), when we perform decoding analyses within format, we can identify a similar off-diagonal pattern shown as that found in Karami et al.. As such, it appears to be a difference between the within- and cross-format decoding of number that underlies this change in temporal structure. Indeed, when we run the same, within-format analyses in our current MEG data, we also observe a stronger off-diagonal pattern, particularly in the first 400ms after stimulus presentation:

**Author response image 2.** Within and Cross-Format Decoding of Empty Sets vs Dot Patterns and Symbolic Zero vs Positive Numbers.

Other factors may play a role in differences between the studies. Stimulus presentation was twice as long in Karami et al’s study compared to our experiment. Only one stimulus format was used by Karami et al, increasing the number of repetitions per non-symbolic numerosity and thus statistical power. Indeed, in Barnett and Fleming (2024), when we used a slower non-symbolic task and restricted the experiment to include two tasks (compared to three in the present study), we can identify non-symbolic numerosity decoding which is very similar to that of Karami et al.

We agree with the reviewer that the temporal structure of domain-general and domain-specific representations deserves further study, and in our previous Current Biology paper we speculated on some reasons for these differences. In the current manuscript for space reasons, we prefer to restrict the discussion to issues specific to the question of representations of absence.

R1C5. Throughout the manuscript, the authors appear to treat non-symbolic numerosity as a conceptual representation and contrast it with perceptual absence. I find this interpretation insufficiently justified. A substantial body of behavioral (Anobile et al., 2013; Cicchini et al., 2016) and neuroimaging (Piazza et al., 2004; Castaldi et al., 2019; Karami et al., 2025) research has argued that non-symbolic numerosity is represented as a perceptual attribute extracted relatively early in the visual processing hierarchy, even if its precise computational origin remains debated. Consequently, it is not immediately clear why non-symbolic numerosity should be regarded as a conceptual representation comparable to symbolic number or the concept of zero. This distinction is important because it directly affects the interpretation of the negative cross-decoding results. If both perceptual absence and non-symbolic numerosity are primarily perceptual representations, the absence of cross-decoding cannot be taken as evidence that perceptual and conceptual absence are represented differently. Rather, it may simply indicate that these two perceptual representations encode different visual attributes. I therefore encourage the authors to clarify their theoretical position regarding the representational status of non-symbolic numerosity and to discuss how their interpretation relates to influential theories of numerical cognition that conceptualize non-symbolic numerosity as an early perceptual representation rather than an abstract conceptual one.

This is a critical point and we thank the reviewer for encouraging us to clarify our position here. We agree that there is good evidence to suggest that the extraction of non-symbolic numerosity depends in part on perceptual representations. Indeed, we did not intend for our non-symbolic numerosity stimuli to strictly probe for conceptual representations. Rather, the primary aim of the experiment was to test for representations of absence across numerical and non-numerical domains, with detection of low-level visual stimuli being most clearly perceptual, and symbolic numerals most clearly conceptual. To ensure clarity here, we have reworded several passages in the abstract and introduction and introduced a new paragraph in the discussion:

Abstract (p. 2):

“The ability to represent absences is fundamental to human perception and cognition. Sensitivity to non-numerical perceptual absences depends on inferring a lack of sensory evidence, enabling the recognition of a lack of environmental resources or other objects. In turn, encoding numerical absence as a concept – in the form of the number zero – has enabled a multitude of technological and mathematical advances.”

“In contrast, we show that these representations of numerical absence are distinct to those evoked by non-numerical perceptual absences.”

Introduction (p. 5):

“Neural representations of the number zero show similarly distinct properties. Like non-numerical perceptual absences, numerical absences are actively represented in higher-order brain regions of non-human primates (Okuyama et al., 2015; Ramirez-Cardenas et al., 2016) and crows (Kirschhock et al., 2021). “

“Finding that conceptualisation of zero overlaps with representations of sensory absence in a detection task would offer support to accounts that propose the evolutionary and developmental trajectory of zero concepts emerge from a more basic ability to track an absence of sensation (Nieder, 2016; Barton, 2020). On the other hand, finding that neural representations of non-numerical and numerical absences are distinct would support an extension of a boundary between perception and cognition into the domain of absence perception (Block, 2023; Firestone & Scholl, 2016).”

Discussion (p. 17):

“In order to interpret the lack of generalisation of absence representations between the detection and non-symbolic numerosity tasks, it is useful to consider the representational status of non-symbolic numerosities. A large body of both behavioural (Burr et al., 2018; Burr & Ross, 2008; Cicchini et al., 2016) and neuroimaging (Castaldi et al., 2019; Park et al., 2016; Piazza et al., 2004) work has suggested that non-symbolic numerosities are represented as a primary perceptual attribute in the visual system, on a par with properties such as contrast, size, and orientation. In this light, a lack of absence generalisation between the detection and non-symbolic numerosity tasks would not delimit a boundary between perceptual and conceptual absences, but rather support a boundary between numerical and non-numerical perceptual absences. Such a divide may be due to perceptual-numerical absence representations requiring additional processes mapping non-symbolic empty sets onto a neural number line (Barnett & Fleming, 2024; Kutter et al., 2024), which is not required – and, as the present results seem to indicate, not instantiated – by non-numerical perceptual representations. “

R1C6a: I have two related concerns regarding the discussion of Paul et al. (2022). First, I think it would be helpful to describe more explicitly what was measured in that study. To my understanding, Paul et al. quantified the aggregate Fourier power (AFP) of the stimuli.

We agree that the methodology of Paul et al. (2022) could be made clearer. We have edited the following passage introducing the study (p. 13):

“One possibility is that the spatial frequency profile of non-symbolic dot patterns covaries with numerosity and can drive early visual responses to non-symbolic stimuli in numerical tasks. This has been shown in previous work where the aggregate Fourier power of dot-stimuli correlates with numerosity (Paul et al., 2022).”

R1c6b: Moreover, AFP has primarily been discussed in the context of dot arrays with constant dot size within each stimulus. In the current manuscript, it is not entirely clear from the Methods whether dot sizes vary within displays. I therefore encourage the authors to explicitly describe how dot sizes were generated and varied across stimuli. If AFP is correlated with numerosity in the present stimulus set, it would also be helpful to explain how the analyses dissociate neural representations of numerosity from those potentially driven by AFP.

We apologise for omitting this information in our initial submission. To be clear, dot size did not vary within each stimulus display. We have now made this explicit in the Methods section describing stimuli (p. 21):

“Low-level visual properties of the stimuli (total dot area, density, luminance) did not covary with numerosity (Figure S1). Total dot area was controlled for by systematically reducing the size of the dots as the number of dots increased, such that the total number of pixels included in a stimulus were constant across numerosities. Dot sizes were held constant within each individual stimulus display.

R1C6c: Second, I am not entirely convinced by the argument that training a classifier to distinguish Hits from Correct Rejections is sensitive to aggregate Fourier power. It would be helpful if the authors could explain more explicitly why this decoding contrast should be expected to be sensitive to AFP. As currently written, the logical connection between the AFP hypothesis and the proposed control analysis is not entirely clear.

We thank the reviewer for the opportunity to clarify why we ran this control analysis. The purpose of this analysis was to demonstrate that low-level visual properties could in principle drive spurious generalisation between the detection and non-symbolic task. Showing this is important for two reasons. First, it provides a potential explanation for the small cluster of generalisation between the detection and non-symbolic tasks. Second, it bolsters our main analysis (Figure 2), by showing that even in the context of residual stimulus-related drivers of cross-decoding, we can still find very strong evidence for an absence of shared representations between the two tasks.

More generally, the grating stimuli and empty noise patches naturally vary in a variety of low-level visual properties, not only of their spatial frequency profiles, but their contrast, density, and so forth. Even though our main analysis controlled for stimulus presence in the detection task (equal number of stimulus present trials in ‘seen’ and ‘unseen’ responses), we reasoned that it was still possible that these stimulus features may covary with participants’ decisions on a trial-by-trial basis. It would therefore be possible for our Presence vs. Absence decoder to pick up on a difference in low-level visual features, such as spatial frequency, AFP, contrast, etc.

To ascertain whether any of these low-level visual properties could in principle drive spurious generalisation between the detection and non-symbolic task, we performed an analysis where a decoder was trained to classify empty noise patches from high-contrast gratings (Misses vs. Hits), thus enabling it to rely on differences in stimulus information when classifying the neural data. We then tested this on neural data collected during Empty Set vs. Non-Zero Dot stimuli, reasoning that any above-chance decoding would reflect low-level visual similarities between the two stimulus sets that were distinct from the representations of absence we were interested in. We feel comfortable making this assertion because, in our main analysis, we controlled for stimulus visual information, and saw very little generalisation. Thus, any additional cross-generalisation in the present analysis must be due to the added stimulus confounds.

To make this reasoning more explicit in the manuscript, we have reworded the Results section that motivates and introduces this control analysis (p. 13-15):

“Taken together, our analyses indicate strong evidence (as derived from BFs) that neural representations of perceptual and numerical absence can be robustly identified in patterns of MEG activity, and that these representations are distinct rather than shared. As noted above, the one exception to this pattern was a small but significant cluster of activity around 300ms that indicated some shared representation of non-symbolic and perceptual absences (Figure 2A; Figure 2B). We considered that this exception may have been driven by uncontrolled shared variance in lower-level visual features of our detection and empty-set stimuli. One possibility is that the spatial frequency profile of non-symbolic dot patterns covaries with numerosity and can drive early visual responses to non-symbolic stimuli in numerical tasks. This has been shown in previous work where the aggregate Fourier power of dot-stimuli correlates with numerosity (Paul et al., 2022). Additionally, stimuli containing dots naturally possess higher contrast than blank empty-set stimuli. Crucially, similar visual properties may also characterise our detection stimuli, with spatial frequency and contrast profiles differing between grating stimuli and noise patches. It is possible that these, or other, stimulus-related visual confounds may contribute to the brief cluster of generalisation observed between perceptual and non-symbolic stimuli. By explicitly attempting to decode the physical presence or absence of the grating (which up until now was controlled for in our analysis) we aimed to further characterise this component of visually-driven shared variance.

To evaluate this possibility, we trained a decoder to classify Hits vs. Correct Rejections within the detection task (Figure 3A). This decoder should be not only sensitive to the ‘present’ or ‘absent’ decision made by a participant, but also the physical presence (or absence) of a grating. To maximise sensitivity in this analysis, only trials within the supra-threshold contrast level were used to define Hits. We then tested this decoder on empty sets vs. non-zero dot patterns and found a cluster of significant generalisation between 100ms and 300ms, with the peak BF indicating evidence for shared neural signatures across the two tasks (peak log10(BF) = 3.25; time averaged log10(BF) [0.05s, 0.3s] = -0.82; Figure 3B, left). When decoding in the alternative direction (training on empty sets vs. non-zero dot patterns and testing on Hits vs. Correct Rejections), we observed similar patterns of generalisation, with evidence in favour of shared neural signatures between the two stimulus sets (peak log10(BF) = 4.45; time averaged log10(BF) [0.05s, 0.3s] = 0.82; Figure 4B, right). Taken together, this result suggests that visual features may spuriously drive cross-decoding of absence in perceptual and non-symbolic numerical domains, unless stimulus contrast is appropriately controlled for. Indeed, when such properties are controlled for, generalisation across detection and non-symbolic tasks is almost entirely abolished (Figure 2). It is therefore all the more striking that, despite the potential for uncontrolled stimulus driven contributions to cross-decoding, we find strong evidence against shared neural representations of perceptual and numerical absence (Figure 3B).”

**Reviewer #2 (Public review):**

The authors tackle the question of whether conceptual absence is neurally encoded in the same way as perceptual absence. On one hand, the neural bases of perceptual absence have been largely investigated, as exemplified by the study of neural correlates of perception of aware vs. unaware stimuli, and on the other hand, the overlapping neural encoding of symbolic ('0') and non-symbolic (number of dots) formats of numerical absence has been previously established (Barnett & Fleming, 2024). However, the direct comparison of neural representations between numerical and perceptual absences remained to be investigated.

This article fills this gap by designing a Magneto-EncephaloGraphy (MEG) study using multi-voxel pattern analysis (MVPA) and temporal generalization to probe the similarity of neural patterns across the representation of perceptual absence (lack of stimuli), symbolic ('0'), and non-symbolic (number of dots) formats of numerical absence. They confirmed previously obtained evidence for shared neural representation across both formats of numerical absence. They report evidence for an absence of shared representation between both formats of numerical absence on one hand and perceptual absence on the other hand, while controlling for the confounding effect of low-level visual features. Their results overall support the conclusion that conceptual and perceptual absence are neurally encoded in a distinct way and speak in favour of a boundary between the representation of the concepts and the percept of absence.

Major strengths:

(1) Behavioral and neuroimaging results convincingly demonstrate that neural encoding of symbolic and non-symbolic absences is shared and situated on a graded, abstract neural number line, replicating previous results, notably from the authors themselves (Barnett & Fleming, 2024).

(2) They show that neural encoding of perceptual and numerical absence do not generalise across each other, while controlling for spurious confounds due to visual stimuli that are commonly shared in the cases of non-symbolic numerical absence and perceptual absences.

(3) They adequately use Bayesian analysis to distinguish absence of evidence vs. evidence of absence to support their claim.

(4) The interpretation of numerical absence as a representation of the concept of "nothingness" is adequate, although it might be further discussed by distinguishing the concept of "zero" on a number line from the concept of nothingness and that of an empty set (Nieder, 2016).

(5) The discussion about development and metacognition paves an interesting road for further investigation on the acquisition of the concept of zero, especially in light of debates on the progressive development of metacognitive abilities in children (Goupil & Kouider, 2019).

(6) Data and code are published with open-source access, allowing the community to further investigate the points as major weaknesses evoked below, if desired.

We thank the reviewer for their careful reading of our manuscript and this positive appraisal of features of our work. Below we provide point-by-point responses to the reviewer’s comments and suggestions.

Major weaknesses:

R2C1: Interestingly, restricting the neural decoding method to the alpha band shows distinct representations across formats of numerical absence. This begs for providing more details on how neural representations of perceptual, symbolic, and non-symbolic absences differ at the source and frequency level and to report the decoding weights to better assess what drives the performance of the neural decoding algorithm in each case and whether they overlap with each other.

We thank the reviewer for encouraging us to explore this question. We conducted new analyses to source-localise the decoder weights for the three different decoders (Perceptual Misses vs. Perceptual Hits; Empty Sets vs. Dot Stimuli; Symbolic Zero vs. Positive Numerals). Since the decoder used was a binary LDA, the appropriate transformation of classification weights for localisation purposes simplifies to a subtraction of mean activity in one class from that of the other class (Haufe et al., 2014; NeuroImage). As such, we computed the difference in mean source-level activity between each of the two classes per decoder. We have added Supplemental Figure 4 to the Supplemental Figures to highlight the brain regions the classifier relied upon to decode representations of absence.

P.31:

“Both FieldTrip’s template single shell head model and its standard volumetric grid (8mm resolution) were warped to participants’ individual fiducial points, generating a subject-specific forward model aligned in MNI space. Source reconstruction was performed using a linearly constrained minimum variance (lcmv) beamformer which applies spatial filters to the MEG data to generate source-level time courses. To reduce the impact of noise on the source estimates, we used a regularization parameter of lambda = 5%. For each task, spatial filters were calculated by combining the leadfield matrix with the data covariance matrix across all stimuli and the timepoints 100 – 450ms. These spatial filters were then applied to absence trials and presence/non-zero trials separately, generating reconstructed maps of source activity for different trial types. The maps can then be contrasted to reveal the source-level decoding weights associated with binary LDA decoding (Haufe et al., 2014; Supplemental Figure 4).”

R2C2: Task-demands between perceptual (present vs absent) and numerical (lower vs higher) are different, raising concerns about whether these aspects of experimental design could drive, at least partially, the shared representational patterns across numerical representation of absence and their distinction from perceptual representation of absence.

We agree with the reviewer that our motivation for this design should be clearer in the manuscript. As we explained in response to R1C1, we deliberately chose to employ distinct perceptual and numerical tasks because we wanted to create a strict test of whether absence representations generalise. Having two closely-matched tasks would have led to a converse difficulty in interpreting any successful cross-decoding because it would have been difficult to ascertain whether generalisation occurred because of a true domain-general representation of absence, or whether it was driven by other features of task design and stimulus-response contingencies.

We appreciate though that our null findings require us to ensure that this design choice has not resulted in a type 2 error. We think this is unlikely for several reasons: first, we focus on stimulus-triggered responses, and within each task, we are able to robustly decode stimulus-triggered absence; second, in our previous work we obtained robust generalisation of absence representations even when tasks are distinct, a finding we now replicate here with more closely matched tasks; and finally, our control analyses of colour do show positive generalisation across task format.

As we noted in response to R1C1, to make the motivation behind this design choice clearer to the reader, we have modified the following passage (p. 6):

“Importantly, we used numerical and perceptual tasks that differed with respect to their structure and temporal dynamics. This ensured that any successful cross-decoding of absence could not be attributed to low-level or structural similarities between tasks, and instead would indicate a domain-general representation of absence. This approach builds on our previous work in which we identified a shared representation of numerical absence across non-symbolic and symbolic stimuli that generalised across task formats (Barnett & Fleming, 2024).”

However, the reviewer is correct that this approach does not allow us to test whether unmeasured variables (such as cognitive load, decision making, etc) could interfere with cross-decoding, thus eliciting false negatives in our analyses. To elaborate on this limitation, and to suggest possible extensions to this work that could begin to help resolve this issue, we have added the following passage to the discussion (p. 19):

“The tasks we employed in this study were dissimilar by design. This offered a strict test of the hypothesis that absences share representational currency across perceptual and numerical domains, because successful cross-decoding could not be artificially inflated by low-level task similarities across domains (Barnett & Fleming, 2024). However, this approach also carries the risk that task dissimilarity reduced our sensitivity to shared representations of absence. We think this is unlikely for several reasons: first, we focus on stimulus-triggered responses, and within each task, we are able to robustly decode stimulus-triggered absence representations; second, in our previous work we obtained robust generalisation of absence representations even when tasks are distinct, a finding we now replicate here with more closely matched tasks (Barnett & Fleming, 2024); and finally, our control analyses of colour do indicate positive generalisation across task format. Future work could explore the relationship between absences across domains using factorial designs that vary both domain and task independently, thus exposing possible interactions between absence representations and task context.”

R2C3: The same argument can also be raised for the way that the neural decoders of perceptual and numerical absences are trained and tested. Both formats of numerical absence are built using the same procedure (zero vs. rest) and differ from the way the decoder is built for perceptual absence (hits vs misses), which might possibly drive the difference observed here.

We are confident that the neural decoders for perceptual and numerical absence are near-identical and highly unlikely to drive the lack of cross-decoding. The essential features of the neural data fed into the decoders are matched across tasks. These two classification schemes – [zero vs. rest] and [hits vs. misses] – are both balanced with respect to the classes of interest. There are an equal number of ‘non-zero’ trials in the [zero vs. rest] decoder, and an equal number of each of the non-zero numbers within the set of non-zero trials. Similarly, the [hits vs. misses] decoder is balanced between hits and misses, and is balanced within each stimulus intensity level. Thus, both decoders are trained and tested on similarly balanced and matched stimulus sets. We note also that the cross-validation scheme used to train the decoders was identical across the two classification schemes. The primary difference between the two decoding schemes is the type of absence that they are seeking to decode.

R2C4: It is thus unknown whether the claim supporting the evidence of absence holds as long as other counterfactual hypotheses that might drive these results are not excluded, such as the nature of the decoded features, the effect of task demands, or the way the neural decoder is trained, as mentioned above.

We fully agree with the reviewer that it is important to mention and discuss the different features that may have contributed to our failure to cross-decode absence representations. We hope that by responding to R2C2 and R2C3 above, we have satisfied the reviewer’s concerns regarding these alternative explanations.

Overall, I was pleased by the quality of the methods and the clarity with which the question of the boundary between cognition and perception is addressed for the case of numerous and perceptual absence. While the methods used are well established in the field, the choice and rigor of their analysis and the controls provided stand as a convincing methodology to test their hypotheses, although they do not fully exclude alternative interpretations nor explore the wider extent of possibilities that may provide exhaustive evidence for showing that perceptual and numerical absences are distinctly encoded in the brain.

This work will be of great appeal to neuroscientists interested in comparing the representation of percepts and concepts across different formats, to psychologists interested in the origin of number representation, and to philosophers interested in debates on the boundary between cognition and perception.

**Recommendations for the authors:**

**Reviewer #1 (Recommendations for the authors):**

(1) In your previous work (Barnett & Fleming, 2024), you reported a curved representation of both symbolic and non-symbolic numerical representations in MDS space. Similarly, Karami et al. (2025) observed a curved representational geometry in both parietal fMRI activity and later MEG representations. Through simulations, they argued that a curved MDS geometry is consistent with numerosity-tuned population codes, whereas a more linear organization is expected from summation-code representations. Given these converging observations, I wonder whether you can examine the geometry of the representational space and whether it evolves over time from a more linear to a more curved organization. It could provide additional insight into the underlying coding scheme. Even if no such transition is observed, reporting the MDS geometry would strengthen the manuscript by helping readers relate the present findings to previous work and by providing a richer characterization of the neural representations beyond decoding accuracy alone.

We agree that the coding scheme and representational space for numerosities is an interesting question. As the reviewer points out, these analyses were a focus of our previous Current Biology paper, and we now explore similar plots in the current data.

P. 9:

“Multidimensional scaling of sensor-level data similarly revealed numerosities to be arranged along a single dimension encoding numerical magnitude that is shared between both numerical formats (Supplementary Figure 2). These analyses confirm a graded representation of numerical absence that is evoked by both non-symbolic empty sets and symbolic zero (Barnett & Fleming, 2024), and establish that we are able to robustly recover neural representations of numerical absence in our paradigm using MEG.”

(2) In Results, clarify whether RTs (figure 1E) are 0.32 s for absent and 0.28 s for present (lines 144-146) - it currently reads "0.28 ms" which must be a typo (should be s).

We have fixed the typo in lines 144-146:

“Replicating previous findings, participants took longer to report stimulus absence (mean RT = 0.32s, SD = 0.14s) compared to stimulus presence (mean RT = 0.28s, SD = 0.11s, t(28) = -3.0176, p = .0054, Figure 1E).”

(3) When describing Bayes factors, the text could define what "time averaged log10(BF)" means in simpler terms, to help readers interpret Figure 2B.

We thank the reviewer for this suggestion. To make this clear to the reader, the manuscript now states (p. 11):

“To allow us to obtain evidence for or against shared representations of numerical and perceptual absence, we computed Bayes factors (BF) – with positive BFs indicating evidence in favour of generalised representations, and negative BFs indicating evidence in favour of distinct neural representations. To assist in visualisation, we plot log10 transformation of BFs such that BFs supporting either distinct or generalised representations are symmetric around zero. Interpretation of log10 BFs follows standard convention for interpreting BFs, with log10(BF) in the range [0, ±0.5] offering weak evidence, [±0.5, ±1] moderate evidence, [±1, ±1.5] strong evidence, [±1, ±2] very strong evidence, and [±2, ±inf] being decisive (Jeffreys, 1998). To report log10 BFs in the text, we average BFs over the 0.2s – 0.8s time window shown in Figure 2C to report a time-averaged log10 BF. In our plots, we indicate the timepoints when evidence for either hypothesis exceeds the threshold for strong evidence.”

**Reviewer #2 (Recommendations for the authors):**

I enjoyed how clear the question of the boundary between cognition and perception was formulated and how the use of temporal generalisability elegantly provides a convincing way to test it.

To address the first major weakness, I would like to suggest providing the decoding features, and how they relate to the neural correlates of absence to strengthen the claim and reassure both reviewers and readers that the MVPA analysis does not pick up on other neural features than those previously established in relation to the neural encoding of absence in each of those formats.

We have now added a supplementary figure displaying the decoding weights in source space in response to the reviewer’s comment R2C1.

Although Figure 2C (top) provides a nice argument in favour of the MVPA actually picking up information related to numerosity, it might also be theoretically explained by the task demands of identifying which number is higher. Although I am aware that the decoder was trained on absence vs rest, the dimension tested (distance from zero) might be confounded with the task to compare which number is higher, while an orthogonal task to numerosity would exclude such potential confound of a task effect on the way zero is represented in the brain. The contextual effect of the task on the neural encoding of numerical absence is perhaps far-fetched, but it seemed to me to be a reasonable concern.

We agree that similarities in task design between the two numerical tasks may contribute to the generalisation of across format observed in our decoding analysis. Indeed, this is precisely why we designed a perceptual (detection) task that shared minimal structure with the numerical tasks, to ensure that task demands would not confound interpretation of findings of absence generalisation (see also our responses to reviewers R2C2 and R1C1). Similarly, in our previous paper in Current Biology (2024) we employed tasks with distinct formats, and found similar results. We therefore think that it’s unlikely that the similarity in task format here is the primary driver of cross-format generalisation, but we cannot rule it out. We believe that our answer to R2C2 addresses this concern and makes the reasoning behind our design choice explicit to the reader.

I became aware, including in my own experiments, that the choice of parameters might be crucial in interpretations of decoding when it comes to neuroimaging results. One of the strengths of MVPA is to be data-driven, reducing the potential selection bias, but it also raises other concerns due to the poor controllability of the relevant features this technique grasps depending on the task and experimental set-up. This begs the question whether the results here obtained are generalisable, taking into account other potential confounds and at least acknowledging such potential limitations in the discussion.

We thank the reviewer for this comment, and agree that chosen parameters can sometimes influence neuroimaging decoding results. To better justify why we chose the parameters we did, we have added the following sentence to the Methods (p. 29):

“All decoding hyperparameters were chosen to match those of our previous work on numerical absence decoding (Barnett & Fleming, 2024), such that results would be directly comparable across the two studies.”

My overall uptake is that the authors make an inference to the best explanation by having designed the most convincing analysis possible to provide a crucial test on the distinction between the neural representation of perceptual and conceptual absence. They rightly exclude the false positive case of low-visual features driving the similarity between perceptual and non-symbolic representation. However, providing the evidence of absence also needs to address possible cases of false negatives that might be a result of their experimental and analytical choices.

Even if proving the evidence of absence for this reason is inherently difficult, if not almost impossible to achieve, it seems reasonable that further insights such as for example comparing the neural correlates of absence as done by the authors in their Current Biology paper based on empirical findings and the possible limitations of their approach might be addressed at least in the discussion to frame their conclusion in regard to the context of their experiment and analysis. This would enrich the paper by providing better hindsight about the origin and the evidence at hand on what might drive the reported distinction in the neural representation of perceptual vs. numerical absence.

We thank the reviewer for their careful reading of our paper, which prompted further considerations of the limitations of different design choices. We have now included new sections of the discussion to ensure these are appropriately highlighted to the reader.

I would like to thank the authors and editors for this opportunity of reviewing this stimulating paper, enriching our perspective on the important debate on the boundary of perception and cognition.

Matthieu Koroma
